# The heterogeneity of signaling pathways and drug responses in intrahepatic cholangiocarcinoma with distinct genetic mutations

**DOI:** 10.1038/s41419-023-06406-7

**Published:** 2024-01-11

**Authors:** Yangyang Feng, Ming Zhao, Lijian Wang, Ling Li, Josh Haipeng Lei, Jingbo Zhou, Jinghong Chen, Yumeng Wu, Kai Miao, Chu-Xia Deng

**Affiliations:** 1 Cancer Center, Faculty of Health Sciences, University of Macau, Macau SAR, China; 2 Zhuhai UM Science & Technology Research Institute, Zhuhai, Guangdong China; 3 Centre for Precision Medicine Research and Training, Faculty of Health Sciences, University of Macau, Macau SAR, China; 4 Department of Gastroenterology, Clinical Medical College and The First Affiliated Hospital of Chengdu Medical College, Chengdu, China; 5 MOE Frontiers Science Center for Precision Oncology, University of Macau, Macau SAR, China

**Keywords:** Translational research, Targeted therapies, Liver cancer, Cancer models, Tumour-suppressor proteins

## Abstract

Intrahepatic cholangiocarcinoma (ICC) is the second most common malignancy among primary liver cancers, with an increasing overall incidence and poor prognosis. The intertumoral and intratumoral heterogeneity of ICC makes it difficult to find efficient drug therapies. Therefore, it is essential to identify tumor suppressor genes and oncogenes that induce ICC formation and progression. Here, we performed CRISPR/Cas9-mediated genome-wide screening in a liver-specific Smad4/Pten knockout mouse model (*Smad4*^*co/co*^*;Pten*^*co/co*^*;Alb-Cre*, abbreviated as SPC), which normally generates ICC after 6 months, and detected that mutations in *Trp53*, *Fbxw7*, *Inppl1*, *Tgfbr2*, or *Cul3* markedly accelerated ICC formation. To illustrate the potential mechanisms, we conducted transcriptome sequencing and found that multiple receptor tyrosine kinases were activated, which mainly upregulated the PI3K pathway to induce cell proliferation. Remarkably, the *Cul3* mutation stimulated cancer progression mainly by altering the immune microenvironment, whereas other mutations promoted the cell cycle. Moreover, Fbxw7, Inppl1, Tgfbr2, and Trp53 also affect inflammatory responses, apelin signaling, mitotic spindles, ribosome biogenesis, and nucleocytoplasmic transport pathways, respectively. We further examined FDA-approved drugs for the treatment of liver cancer and performed high-throughput drug screening of the gene-mutant organoids. Different drug responses and promising drug therapies, including chemotherapy and targeted drugs, have been discovered for ICC.

## Introduction

Cholangiocarcinoma (CC) accounts for 10–15% of all primary liver cancers, except for hepatocellular carcinoma (HCC), and its overall incidence and mortality rates have increased in recent years [1]. CC is a biliary epithelial tumor divided into three subtypes according to its anatomical site: intrahepatic CC (ICC), perihilar CC (PCC), or distal CC (DCC) [2]. ICC is localized in the periphery of the second-order bile ducts inside the liver, whereas both PCC and DCC are located outside the liver. Mixed HCC-ICC tumors share features of HCC and ICC and present aggressive tumor growth and poor prognosis [3]. ICCs are malignant tumors and most patients are diagnosed at an advanced stage [4]. The intertumoral heterogeneity of ICC at the genomic and epigenetic levels makes it difficult to find efficient therapies. Identifying the complex mechanisms of ICC formation and progression is crucial for the development of new therapeutic strategies.

Numerous studies have revealed genetic and epigenetic changes in ICC using integrative analytical tools. Key regulators have been shown to be mutated in CC and are related to different signaling pathways [3]. However, among patients with genomic alterations, most of the identified genes had a low frequency (<5%), suggesting that many of them might be passenger rather than driver mutations during ICC formation and progression [5]. However, in genomic profiling of 200 human CC specimens, the majority (61.5%) did not show any known mutations, suggesting that many cancer driver mutations remain unidentified [5].

Smad4 is a tumor suppressor gene with frequent mutations in ICC [6]. Loss of Smad4 is lethal to embryonic development in mice; thus, a Smad4 conditional knockout mouse model was generated to examine its role in postnatal development [6]. Pten is another multifunctional tumor suppressor gene that is commonly lost in a wide range of cancers; it is a dual-specificity protein phosphatase that antagonizes PI3K activation and AKT phosphorylation [7]. A previous study demonstrated that conditional knockout of either Smad4 or Pten in hepatocytes failed to develop tumors in the liver within 10 months, but visible tumor foci could be observed in Smad4^co/co^Pten^co/co^Alb-Cre (SPC) mice at approximately 4 months [8]. Most SPC mice presented ICC with foci sizes larger than 0.2 cm after 6 months and most likely generated ICC at 8-10 months [8].

To identify additional drivers that accelerate ICC formation, we previously performed CRISPR/Cas9-mediated genome-wide screening targeting 20,611 genes in SPC mice and identified Cul3 as a tumor suppressor, whose disruption accelerated ICC formation by inducing inflammation in the liver [9]. In addition to Cul3, our analysis also detected sgRNAs for many other genes that appeared with high frequency in association with ICC formation. In this study, we further analyzed our top candidates and found that a deficiency in Trp53, Fbxw7, Inppl1, or Tgfbr2 could significantly enhance ICC formation in SPC mice, and these genes were associated with several distinct signaling pathways. We also conducted high-throughput drug screening of organoids derived from ICCs associated with these mutations, providing potential therapeutic strategies for their treatment.

## Methods

### Cells

The ICC cell line, 273cc, was isolated from SPC mice. For in vitro cell culture, cells were grown in complete F-medium as previously described [10]. Cell proliferation was measured using the Alamar Blue assay, according to the manufacturer’s protocol. Briefly, cells were incubated in 10% v/v Alamar Blue (Sigma, R7017) at 37 °C for 2 h in 96-well plates. At the end of the incubation period, fluorescence was monitored at 590 nm using an excitation wavelength of 530–560 nm. Human CC cell line QBC939 was obtained from Prof. Chundong Yu (Xiamen University, China), and HCCC9810 was purchased from Procell Life Science and Technology.

### Animal experiments

All the mouse experiments were approved by the Animal Care and Use Committee of the University of Macau (Approval no. UMARE-015-2019). All surgical procedures strictly followed the requirements of the Animal Care and Use Committee of the Faculty of Health Sciences at the University of Macau. For in vivo gene knockout, the pX330 vector (30 µg) containing a single sgRNA was dissolved in 1.8 ml of sterile PBS and injected into mice at a constant rate within 5–8 seconds. For subcutaneous injection, CRISPR vector-transfected 273cc cells were implanted into 8-week-old athymic nude mice at a concentration of 1 × 10^7^ cells/ml; each mouse was injected with 100 µL of cells. Tumor volume was measured 2–4 times per week and compared between the different groups of mice (*N* = 6–9 tumors per group). The tumor volume was calculated using the formula *V* = *ab*^2^/2, where *a* is the tumor length and *b* is the tumor width. All the mice were sacrificed after the experiments.

### Vectors

The pX330 (Addgene plasmid #42230) and lentiCRISPR v2 (Addgene plasmid #52961) vectors were digested and ligated with sgRNAs that target candidate genes according to the standard protocol [11, 12]. A mouse genome-scale CRISPR knockout library (#1000000052) was used for screening. The sgRNAs for single gene knockout were selected from the library: MGLibA_56035 (sgRNA1) and MGLibA_56033 (sgRNA2) for *Trp53*; MGLibA_18108 (sgRNA1) and MGLibA_18107 (sgRNA2) for *Fbxw7*; MGLibA_26092 (sgRNA1) and MGLibA_26094 (sgRNA2) for *Inppl1*; MGLibA_53622 (sgRNA1) and MGLibA_53623 (sgRNA2) for *Tgfbr2*; and MGLibA_12475 (sgRNA1) and MGLibA_12477 (sgRNA2) for *Cul3*.

### Lentivirus production and cell infection

The lentivirus was produced by transfecting the lentiCRISPR v2 vector (4 µg), viral packing plasmid delta 8.2 (3.5 µg), and pMD2.G encoding VSV-G (2 µg) into HEK293FT cells in a 10-cm dish using polyethylenimine (PEI) (Polyscience, 24765). The supernatants containing viral particles were harvested at 60 h, filtered through 0.45 μm polyethersulfone (PES) filters, and pooled. The viruses were divided into aliquots and stored at –80 °C for less than 1 month. To generate the CRISPR knockout cell line, the collected viruses were added to 273cc of cells and diluted with F-medium at a 1:1 ratio. After 48 h, the medium containing viruses was washed out and replaced with F-medium containing 1.5 µg/ml puromycin for selection. After 5 days, live cells were collected, and genomic DNA was extracted for gene mutation analysis. sgRNA-targeted fragments were amplified using Q5 High-Fidelity DNA Polymerase. Genetic mutations were confirmed using the Surveyor® Mutation Detection Kit (Integrated DNA Technologies, 706020) and Sanger sequencing. Targeted sequences were amplified using different primers (Supplementary Table S5). The indel frequencies were determined as previously described [13].

### Histology and immunohistochemistry staining

Livers or tumors were harvested, fixed in 10% (v/v) formalin overnight, and embedded in paraffin. Paraffin sections (4 µm) were prepared for staining. The following antibodies were used: AE1 (1:200, Signet Laboratories, 462-01), Hep Par1 (1:100, Dako, M7158), PCNA (1:1000, Abcam, ab18197), Cyclin D1 (1:200, Cell Signaling Technology, 2978), phospho-Stat1 (Tyr701) (1:500, Cell Signaling Technology, 9167), CD45 (1:200, Cell Signaling Technology, 70257), CD3 (1:200, Dako, A0452), F4/80 (1:200, Cell Signaling Technology, 70076), CXCL9 (1:500, Invitrogen, PA5-79115), S100A9 (1:300, Cell Signaling Technology, 73425), and PD-L1 (1:300, Cell Signaling Technology, 13684).

### Western blot analysis

Total protein lysates from tumors were extracted by RIPA lysis buffer supplemented with proteinase and phosphatase inhibitors (Roche Diagnostics, 4693159001). The samples were separated on a 10% SDS‒PAGE gel, transferred to a nitrocellulose membrane (Millipore), and probed with the following antibodies: Fgfr2 (1:1000, Merck, sc-6930), phospho-Fgfr (1:1000, Cell Signaling Technology, 3476), Vegfr2 (1:1000, Cell Signaling Technology, 12599), phospho-Src (Tyr416) (1:1000, Cell Signaling Technology, 2101), β-actin (1:1000, Cell Signaling Technology, 3700), phospho-AKT (Ser473) (1:1000, Cell Signaling Technology, 9271), phospho-AKT (Thr308) (1:1000, Cell Signaling Technology, 13038), and EGFR (1:1000, Cell Signaling Technology, 8339). Full and uncropped western blots are presented in the Supplemental File.

### Tumor organoid culture

Tumors grown in nude or SPC mice were collected, finely minced, and digested in complete DMEM containing 2 mg/mL collagenase IV and 1.5 mg/mL collagenase II for 30 min at 37 °C. Single cells were separated using a 40 µm cell strainer, centrifuged, and resuspended in 50% (v/v) Matrigel (Corning, 354234) diluted in cold advanced DMEM/F12. Matrigel-containing cells were placed in 6-well plates in 25 µL drops and polymerized at 37 °C for 20 min. Two milliliters of F-medium were added to each well after solidification [10].

### Slice culture

Three-dimensional tumor slice cultures (3D-TSCs) were prepared as previously described, with modest modifications [14]. In brief, 300 μm slices were prepared using a Leica VT1200 S vibratome (Leica Biosystems Nussloch GmbH, Germany), embedded in a collagen I-based matrix, and placed in a transwell insert. After solidification, 300 μL complete F-medium was added to the outer dish. In some cases, 10 μg/mL of anti-PD-L1 (BioLegend) was added to the culture medium.

### Drug screening with the organoids

When organoids grew to a sufficient size, the culture medium was removed and replaced with 0.25% trypsin-EDTA. The cells were dissociated from Matrigel at 37 °C by gently pipetting up and down for 5 min. Organoids were passaged 2–3 times until the cell number was sufficient for screening. For high-throughput drug screening, digested single cells were resuspended in a growth medium at a density of 45,000–50,000 cells/mL. Cells were plated onto a solidified collagen layer. Briefly, 10 μL of 1.3 mg/mL collagen I was dispensed into each well of a 384-well microplate. Ten minutes after polymerization at 37 °C, 30 µL of medium containing the cells was dispensed into each well. After 24 h, the drug compounds were added to a concentration series and cell viability was determined after 96 h of incubation using a Celltiter cell viability assay (Beyotime, C0068). The maximum concentration of each compound was 20 µM, and the experimental concentration range was calculated using a 6-point threefold serial dilution of the maximum concentration.

### RNA sequencing and data analysis

Mouse cell line RNA was extracted using the TRIzol reagent. The quantification, integrity, and purity of the RNA samples were examined using Agilent 2100. The integrity values of all the samples were greater than 7. The amount of total RNA in each sample was greater than 400 ng. Messenger RNA was purified using poly T oligo-attached magnetic beads, followed by fragmentation, reverse transcription, second-strand cDNA synthesis, end repair, A-tailing, adapter ligation, size selection, PCR amplification, and purification. The RNA-seq libraries were then ready for sequencing on Illumina platforms, and 150 bp paired-end reads were generated. Raw reads were filtered and cleaned by removing reads containing adapters (*N* > 10%) and low-quality reads, and a quality check was carried out using MultiQC [15]. RNA reads were aligned to the mouse reference genome, GRCm38, using HISAT2 [16] and counted using featureCounts [17]. Differentially expressed genes (DEGs) were identified using DESeq2 [18], and the cut-off criteria were a fold change of no less than 2 and an adjusted *p* value of no more than 0.05. Pathway enrichment analysis was performed using R package clusterProfiler [19].

### Statistics

For group comparisons, two-way ANOVA was used. All values are expressed as the mean ± SEM.

## Results

### Identification of potential ICC tumor suppressor genes in SPC mice

**Fig. 1:**
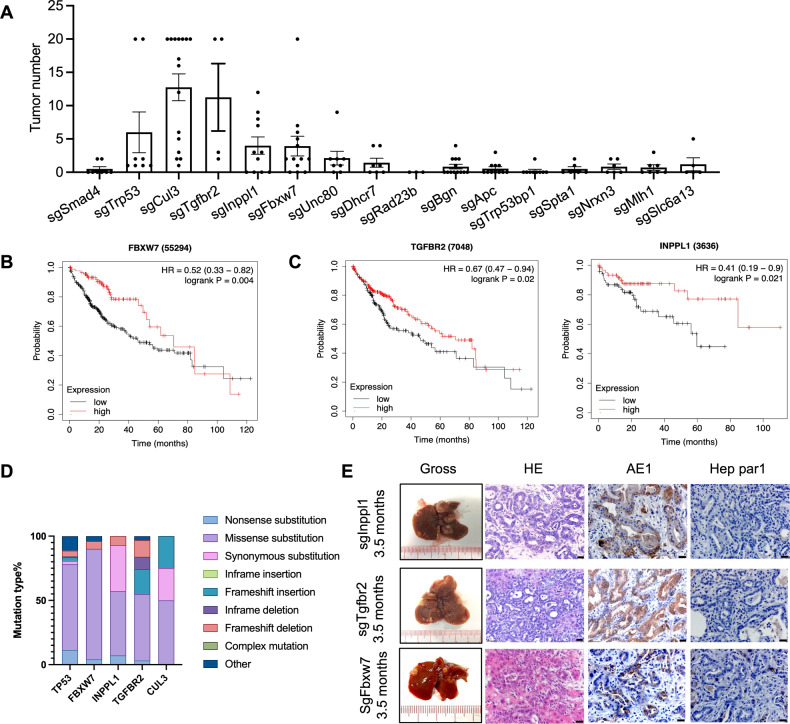
Lentiviral delivery of the CRISPR knockout library identified genes that markedly accelerate ICC formation in SPC mice. **A** Summary of tumor number collected from each mouse. Livers developed tumors at 3–4 months after injection with pX330 containing the corresponding sgRNA. sgSmad4 was included as a negative control. **B**, **C** Kaplan‒Meier survival plots show survival probability with different gene expression. **D** Mutation rates of the *TP53*, *FBXW7*, *INPPL1*, *TGFBR2,* and *CUL3* genes among CC patients based on the COSMIC database. **E** Livers injected with pX330 containing sgInppl1, sgTgfbr2 or sgFbxw7 developed ICC tumors. H&E staining and immunohistochemistry (IHC) staining for AE1 and Hep par1. Scale bar = 20 μm.

In human ICC, mutations in numerous genes have been identified [5] (Supplementary Table S1); however, the nature of these mutations regarding tumorigenesis is largely unknown due to the lack of functional validation. We previously injected a lentiviral CRISPR knockout library into SPC mice and identified several candidate genes via sgRNA enrichment [9]. To identify additional cancer drivers of ICC formation, we conducted functional validation using pX330 vectors containing sgRNA for the 15 top candidate genes through hydrodynamic tail vein (HTV) injection into 4-week-old SPC mice. These genes included *Cul3* [9] and *Trp53* [20], which were previously validated as ICC suppressors, and 13 other genes, whose roles in ICC are currently unknown. At the age of 3–4 months, the mice were sacrificed, and the liver tumors were collected and counted. Mutations in *Trp53*, *Cul3*, and three other genes, including *Fbxw7*, *Inppl1*, and *Tgfbr2*, induced more ICC tumors compared with the control (Fig. 1A, Supplementary Fig. S1A). Next, we analyzed the TCGA human database and found that liver cancer patients with low expression of these genes had significantly shorter survival (Fig. 1B, C; Supplementary Fig. S1B). According to the COSMIC database, the mutation rates of these genes among ICC patients are also high, and most mutations were loss-of-function mutations (Fig. 1D).

To understand the role of these genes, we conducted a comparative analysis of their impact on ICC. Similar to tumors induced by Trp53 and Cul3 loss [9], histological analysis indicated that *Fbxw7*-, *Inppl1*-, and *Tgfbr2*-mutant tumors are ICCs as well, which were located in the periphery of the second-order bile ducts with the phenotype of cuboidal cholangiocytes and were AE1 positive but Hep par1 negative (Fig. 1E). Together, these data provide solid evidence that, similar to Trp53 and Cul3, Fbxw7, Inppl1, and Tgfbr2 also act as tumor suppressors of ICC formation.

### CRISPR targeting genes accelerate ICC proliferation in vitro and in vivo

**Fig. 2:**
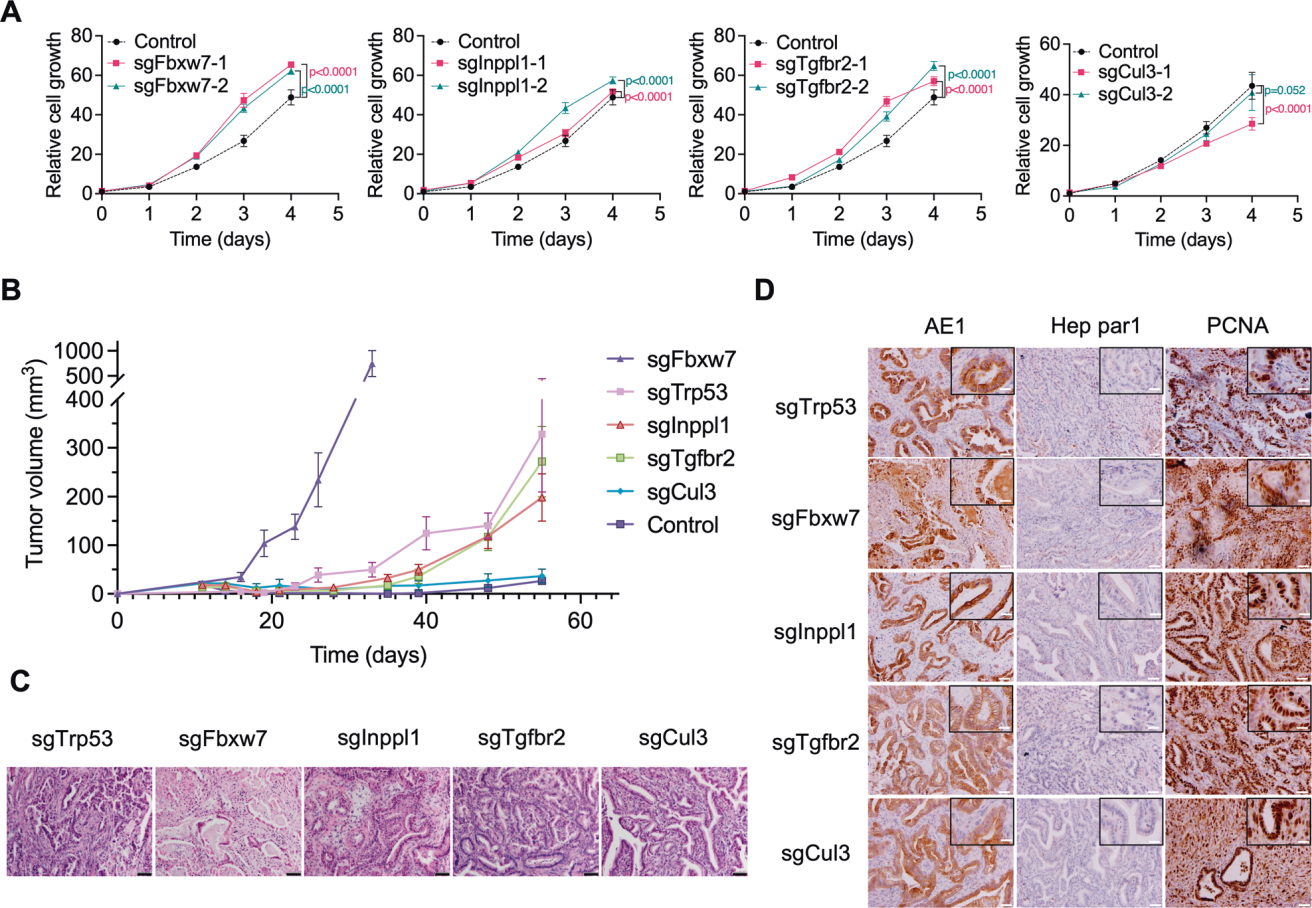
The identified gene mutation promotes cancer progression in vitro and in vivo. **A** Cell proliferation assay in transfected cells. Cell viability was examined with Alamar Blue; the fluorescence intensity was normalized to the values on Day 0. **B** Tumor volume measured at different time points in nude mice bearing 273cc cells transplanted with different sgRNAs. (*n* = 6–9 in each group). **C**, **D** H&E staining and IHC staining for AE1, Hep par1 and PCNA in 273cc cell transplanted tumors with different mutations. Scale bar = 20 μm, inset scale bar = 10 μm.

To validate the functions of these genes in promoting ICC proliferation, we introduced gene mutations into a 273cc cell line isolated from an ICC tumor in SPC mice. Gene mutation sites induced by CRISPR/Cas9 were confirmed using Surveyor assay and Sanger sequencing (Supplementary Fig. S2A, B). Transfection with CRISPR targeting these genes clearly accelerated cell proliferation, except for Cul3, which reduced cell proliferation (Fig. 2A; Supplementary Fig. S2C). Implantation of cells carrying sgRNAs for these genes into nude mice also enhanced tumor formation to varying extents, with sgFbxw7 producing tumors at a much higher rate than those of sgTrp53, sgInppl1, and sgTgfbr2, whereas there was no difference in tumor formation in sgCul3 cells compared to that in the control (Fig. 2B). These data suggest that different mechanisms underlying tumor progression are affected by these genes, particularly Cul3. Our previous study indicated that Cul3 loss-enhanced ICC formation in SPC mice was accompanied by inflammation in the liver [9]. It is conceivable that nude mice do not provide a favorable environment for enhancing sgCul3-induced tumor growth. Indeed, we observed that *Cul3* mutation no longer triggered a robust immune response in nude mice (Supplementary Fig. S2D, E), indicating that the immune microenvironment enhanced ICC formation in the absence of Cul3. Similar results were also found by using human cell line QBC939 and HCCC9810 cells (Supplementary Fig. S2G, H, I).

Further analysis of all these allograft tumors revealed that they exhibited ductal structure and expressed AE1, but not Hep par1, indicating that all allograft models maintained the ICC phenotype (Fig. 2C, D). The cell proliferation-related molecules cyclin D and Ki67 and the immune response-related molecule phospho-Stat1 were highly expressed in all mutant tumors (Supplementary Fig. S2F). Together, these results demonstrate that mutations in *Trp53*, *Fbxw7*, *Inppl1*, *Tgfbr2*, and *Cul3* accelerate tumor growth in SPC mice, whereas mutations in *Cul3* do not enhance ICC growth in nude mice, suggesting a distinct mechanism associated with Cul3-mediated tumor progression.

### The tumor microenvironment varies in ICC tumors with different gene mutations

**Fig. 3:**
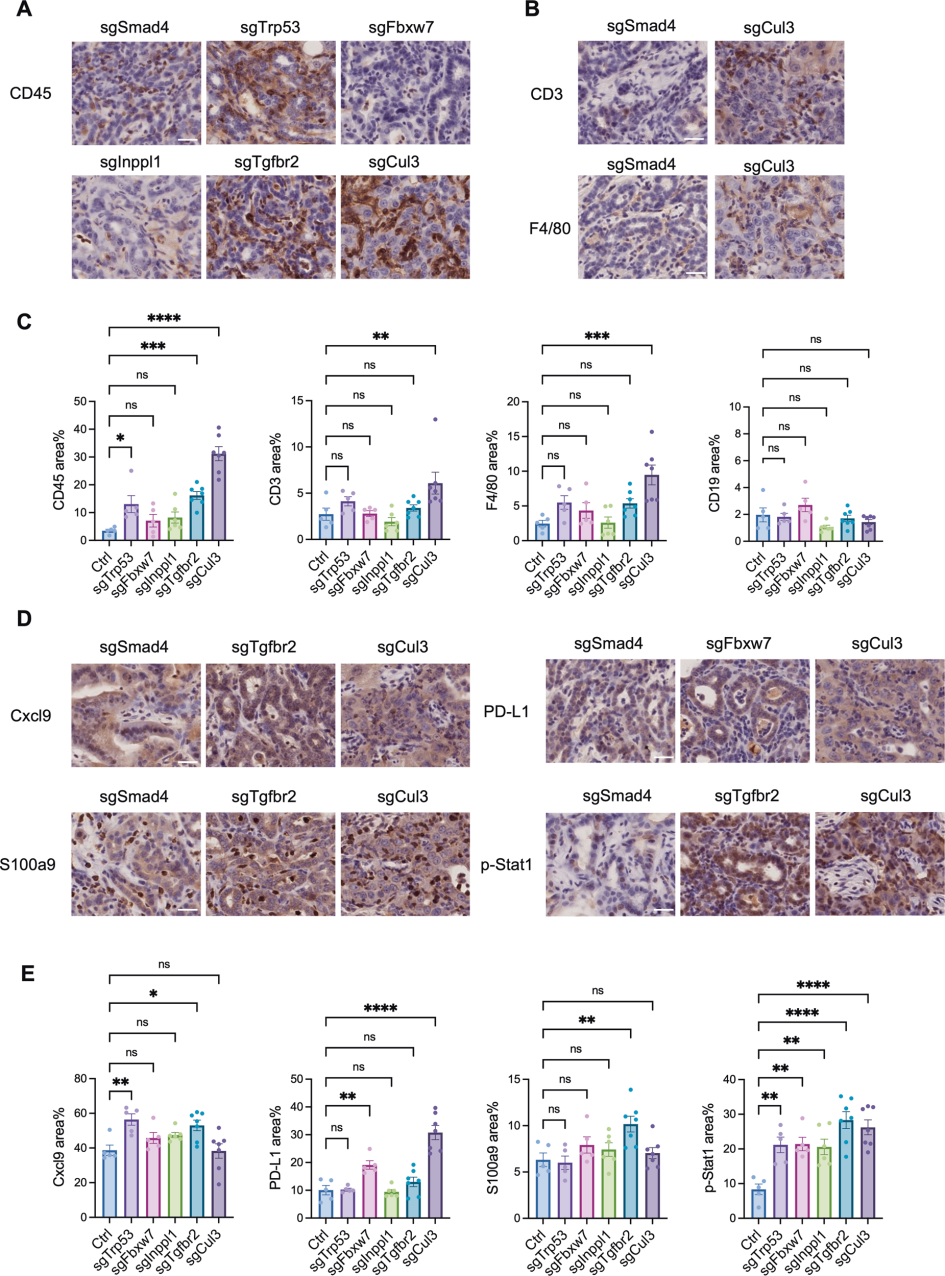
The tumor microenvironment varies in ICC with different gene mutations. **A**, **B** IHC staining of immune cells in ICC tumors developed in SPC mice. CD45 indicates total immune cells, CD3 indicates T cells, and F4/80 indicates macrophages. **C** Statistical analysis of immune markers in tumors, each dot indicates one mouse. sgSmad4 was included as a negative control. **D**, **E** IHC staining and statistical analysis of immune-related molecules in ICC tumors developed in SPC mice. ns: not significant, *p < 0.05, **p < 0.01, ***p < 0.001, ****p < 0.0001. Scale bar = 25 μm.

Thus far, our data imply that immunological changes play a critical role in ICC driven by Cul3 deficiency, whereas their effect on ICC driven by other genes is unclear. To investigate this, we examined the immune microenvironments in ICC tumors with different gene mutations. Similar to previous results, the Cul3 knockout recruited more immune cells, including CD3^+^ T cells and F4/80^+^ macrophages, to the tumor. *Trp53* and *Tgfbr2* mutations also recruited significantly more immune cells (Fig. 3A–C; Supplementary Fig. S3A). The CD3^+^ T cell-attracting chemokine Cxcl9 was more highly expressed in *Tgfbr2*-mutant tumors, whereas the immune inhibitory marker PD-L1 was elevated in *Fbxw7*- and *Cul3*-mutant tumors. S100a9, a marker of myeloid-derived suppressor cells (MDSCs), was highly expressed in *Tgfbr2*-mutant tumors. All gene-mutant tumors exhibited upregulated expression of phosphor-Stat1, indicating strong immune responses (Fig. 3D, E; Supplementary Fig. S3B, C). These results suggest that although genetic mutations can induce ICC, alterations in the immune microenvironment are distinct.

### *Trp53*, *Fbxw7*, *Inppl1*, *Tgfbr2* and *Cul3* gene mutations upregulate receptor tyrosine kinase (RTK) and PI3K signaling pathways in ICC cells

**Fig. 4:**
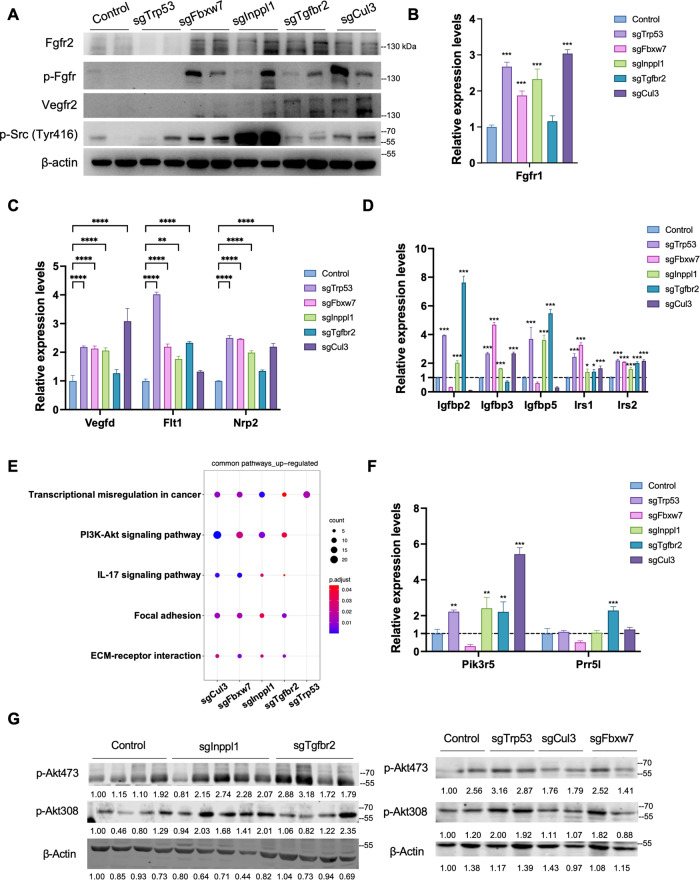
CRISPR/Cas9-targeted gene mutation induced RTK upregulation and PI3K signaling activation. Western blot (**A**) and RNA-seq analysis (**B**–**D**) revealed activation of VEGF, FGFR, and IGFR signaling of each gene-mutant cell (*n* = 3). KEGG enrichment (**E**), RNA-seq analysis (**F**) and western blot (**G**) indicated PI3K pathway activation. The expression levels were normalized to control group. (*n* = 3 for each group). *p < 0.05, **p < 0.01, ***p < 0.001.

Because we had shown that genetic mutations in *Trp53*, *Fbxw7*, *Inppl1*, *Tgfbr2*, and *Cul3* enhanced ICC growth, we then evaluated the corresponding signaling changes in ICC cells by analyzing different levels of gene expression in these gene-mutant cells. To date, the most important signaling pathways identified in ICC are the receptor tyrosine kinase and growth factor-mediated angiogenic signaling pathways, which induce the activation of downstream pathways to modulate cell proliferation, division, or migration [3]. By analyzing gene expression in 273cc cells with different gene mutations, we found that Fgfr2 and phospho-Fgfr were upregulated in *Fbxw7*-, *Inppl1*-, *Tgfbr2*-, and *Cul3*-mutant cells; Vegfr2 was upregulated in *Tgfbr2*- and *Cul3*-mutant cells, and phospho-Src (Tyr416) was strongly elevated in *Inppl1*-mutant cells but weakly upregulated in other cell types (Fig. 4A). Other genes related to FGFR signaling (Fgfr1), VEGF signaling (Vegfd, Flt1 (Vegfr1), and Nrp2), and the insulin growth factor (IGF) pathway (Igfbp2, Igfbp3, Igfbp5, Irs1, and Irs2) were upregulated in cells with different gene mutations (Fig. 4B–D). We also detected FGFR2, VEGFR2 and phospho-SRC (Tyr416) upregulation in mutant human cells (Supplementary Fig. S4C).

Next, we addressed the downstream pathways activated by RTK signaling. We examined the RAS–MAPK and PI3K-AKT–mTOR pathways, which are mainly triggered by RTK signaling, and found that the PI3K-AKT signaling pathway was highly enriched in commonly upregulated genes after gene mutation, whereas RAS–MAPK may be upregulated only in *Trp53*- and *Fbxw7*-mutant cells (Fig. 4E; Supplementary Fig. S4A, B). The pathway-related genes, Pik3r5 and Prr5l, were also upregulated in cells with different mutations. (Fig. 4F). Western blot analysis showed increased phosphorylation of AKT at Ser473 and Thr308 in *Fbxw7*-, *Cul3*-, *Inppl1*-, *Tgfbr2*- and *Trp53*-mutant cells (Fig. 4G, Supplementary Fig. S4C). These results suggest that these gene mutations mainly activate RTKs, such as Fgfr or Vegfr, thus upregulating the downstream PI3K-AKT pathway.

Development-related pathways such as the Notch and Wnt/β-catenin signaling pathways are more active in ICC than in HCC [21]. These pathways are essential for biliary tract development. We next examined genes related to these pathways in transfected 273cc cells and found that both pathways were affected in cells with different gene mutations (Supplementary Fig. S5A, B). Moreover, epigenetic dysregulation, noncoding RNAs (ncRNAs), chronic inflammation, cell cycle, and cell proliferation are regulated in most gene-mutant cells (Supplementary Fig. S6, S5C–G; Supplementary Table S2).

### *Trp53*, *Fbxw7*, *Inppl1*, *Tgfbr2* and *Cul3* gene mutations regulate distinct pathways in ICC cells

Next, we addressed the distinct pathways induced by different gene mutations.

**Fig. 5:**
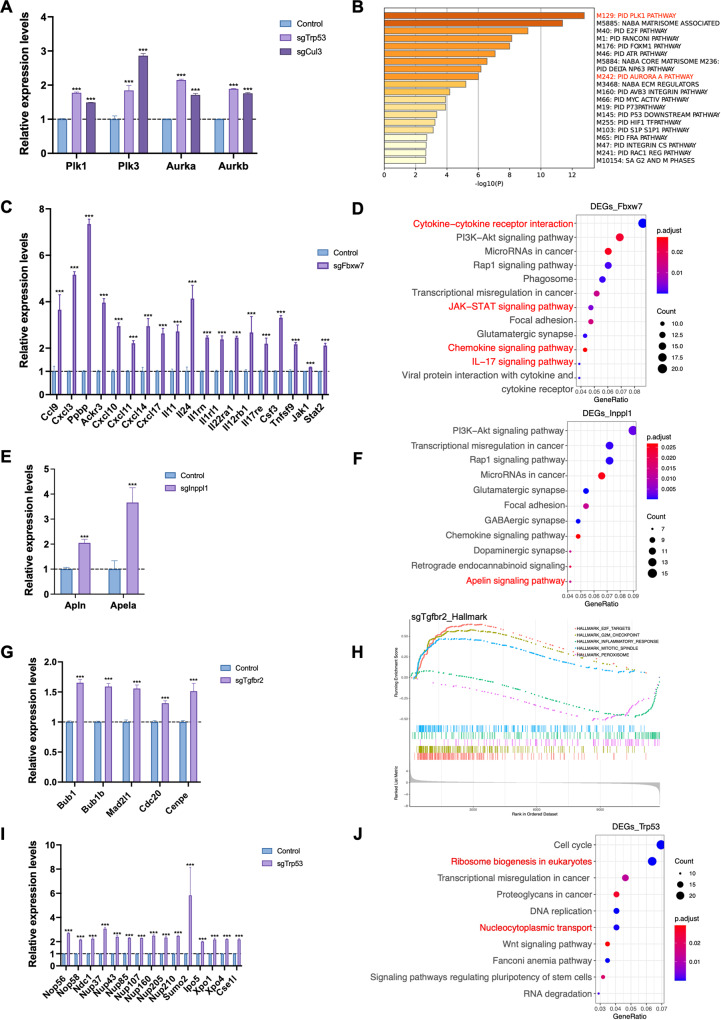
Different pathways regulated by CRISPR/Cas9-induced gene mutation in ICC cells. RNA-seq analysis revealed that sgCul3 (**A**, **B**), sgFbxw7 (**C**, **D**), sgInpppl1 (**E**, **F**), sgTgfbr2 (**G**, **H**), and sgTrp53 (**I**, **J**) induce different pathway changes in CCA cells. ***p < 0.001. The expression levels were normalized to control group. (*n* = 3 for each group).

In *Cul3*-mutant cells, upregulated genes were enriched in cell metabolism. Notably, polo-like kinase (PLK)- and Aurora kinase-related pathways, which play important roles in the cell cycle and mitotic process associated with centrosome and spindle microtubes, were enriched, and the key molecules of these pathways, Plk1, Plk3, Aurka (Aurora Kinase A), and Aurkb (Aurora Kinase B), were upregulated (Fig. 5A, B). Cul3 is well-known regulates the ubiquitination of antioxidants by binding to Keap1 [22]. Indeed, pathways related to reactive oxygen species and cytochrome P450, which are crucial for maintaining redox homeostasis, were significantly enriched based on KEGG enrichment analysis (Supplementary Fig. S6B).

In *Fbxw7*-mutant cells, the most significantly enriched KEGG pathway was cytokine–‒cytokine receptor interaction. Other immune-related pathways, including the JAK-STAT, chemokine, and IL-17 signaling pathways, were also significantly enriched, indicating that downregulation of Fbxw7 in ICC affects immune responses by mediating cytokine signaling pathways (Fig. 5C, D).

Inppl1 is an inositol polyphosphate phosphatase that specifically suppresses the PI3K pathway [23]. Using KEGG analysis, we detected a significantly enriched apelin signaling pathway and upregulation of the key molecules, Apln and Apela, which encode the endogenous ligand for the apelin receptor, thus stimulating downstream signaling, including the PI3K pathway (Fig. 5E, F).

In *Tgfbr2*-mutant cells, we found that the mitotic spindle pathway was enriched based on GSEA hallmark analysis, and tube formation and microtubule cytoskeleton organization were also enriched based on GO. Molecules of the mitotic checkpoint complex were upregulated, which may lead to the inactivation of the spindle assembly checkpoint, which is associated with aneuploidy that can induce cancer (Fig. 5G, H). In *Trp53*-mutant cells, ribosome biogenesis in the eukaryotic pathway and nucleocytoplasmic transport pathway were ranked higher in KEGG analysis (Fig. 5J).

Normal ribosome biogenesis releases E3 ubiquitin-protein ligase Mdm2 to ubiquitinate and degrade p53, and impaired ribosome biogenesis mediates p53 stabilization [24]. Here, we showed that loss of Trp53 regulated ribosome biogenesis via upregulation of small nucleolar ribonucleoproteins, such as Nop56 and Nop58 (Fig. 5I), which are critical for the modification and methylation of ribosomal RNAs. p53 is a shuttling protein and ribosome maturation requires the transport of ribosomal subunits from the nucleus to the cytoplasm. Disruption of the nuclear localization signal inhibits Mdm2 degradation of p53, although it still induces ubiquitination, indicating that p53 is regulated by nucleocytoplasmic transport. However, it remains unclear whether p53 regulates the nucleocytoplasmic transport pathway. Here, we found that in *Trp53*-mutant cells, proteins of the nuclear pore complex and nuclear transport complex were upregulated, demonstrating that loss of Trp53 affects the nucleocytoplasmic transport pathway.

In summary, transcriptome analysis demonstrated that although all these gene mutations promoted ICC, the mechanisms of tumor formation and progression were diverse (Supplementary Table S3).

### FDA-approved liver cancer drugs exhibit different sensitivities to gene-mutant ICC tumors

Clinical drugs for ICC treatment include chemotherapy, targeted therapy, and immunotherapy. Chemotherapeutic drugs that are often used include fluorouracil (5-FU), gemcitabine, platinum agents, and docetaxel. The targeted drugs approved by the FDA are the FGFR inhibitors pemigatinib (Pemazyre), infigratinib (Truseltiq), and futibatinib (Lytgobi); the IDH1 inhibitor ivosidenib (Tibsovo); and the HER2-targeted drug zanidatamab (ZW25). The immunotherapy drug used is durvalumab (a PD-L1 inhibitor) [25–28].

**Fig. 6:**
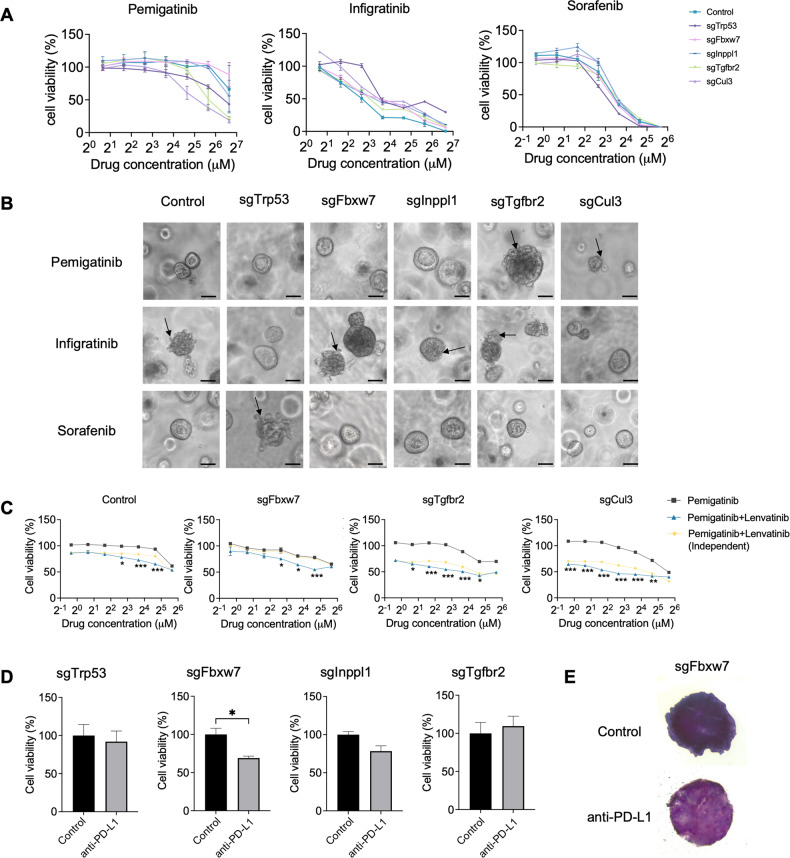
Mutant ICC tumors exhibit different responses to FDA-approved liver cancer drugs. **A** Drug response curves of different drugs in gene-mutant 273cc cells. **B** Morphological differences in different gene-mutant 3D organoids after drug treatment. Scale bar = 50 μm. **C** Drug combination treatment of CRISPR-targeted 273cc cells (*n* = 3). Drug concentrations for pemigatinib range from 50 µM to 0.78 µM (2-fold dilution). Lenvatinib (5 µM) was used for drug combinations. A two-way ANOVA was used for statistical analysis. Independent combination curves were calculated using the Bliss model. **D**, **E** MTT staining of gene-mutant 3D-TSCs after anti-PD-L1 treatment (*n* = 3 for sgTrp53 and sgInppl1 groups, *n* = 4 for sgFbxw7 and sgTgfbr2 groups). *p < 0.05, **p < 0.01, ***p < 0.001.

We first examined the drug effects using ICC organoids. The FDA has approved sorafenib, regorafenib, and lenvatinib for HCC treatment, and pemigatinib and infigratinib for ICC treatment. We found the organoids responded to sorafenib, pemigatinib, and infigratinib in a dose-dependent manner (Fig. 6A; Supplementary Fig. S7A). Among the different gene mutations, *Tgfbr2*- and *Cul3*-mutant cells responded to pemigatinib better than the other mutations, and most cells were sensitive to infigratinib and sorafenib, whereas *Trp53*-mutant cells exhibited greater resistance to infigratinib and sensitivity to sorafenib, which may be due to their lower expression of Fgfr2 (Fig. 4A). Drug effects were also examined using 3D organoid culture (Fig. 6B). As monotherapy typically has severe side effects, combined treatment with lower drug doses has been applied. We examined the synergistic effects of different drug combinations and found that lenvatinib, when combined with other targeted drugs, including pemigatinib and infigratinib, had improved drug effects with lower drug concentrations in most gene-mutant cells (Fig. 6C; Supplementary Fig. S7B). These single drug and drug combinations also worked efficiently in human cells (Supplementary Fig. S7E, F).

Because immunotherapy drugs are increasingly used for treating ICC, we examined the effect of anti-PD-L1 using 3-dimensional tumor slice culture (3D-TLC), which maintains intact tumor microenvironments compared with 3D organoid culture [14]. A previous study demonstrated that anti-PD-L1 treatment combined with sorafenib efficiently inhibits tumor progression in Cul3-deficient tumors [9]. Here, we found that anti-PD-L1 treatment decreased cell viability in *Fbxw7*-mutant tumors, but not in *Trp53*-, *Inppl1*-, and *Tgfbr2*-mutant tumors (Fig. 6D, E; Supplementary Fig. S7C). This indicates that the *Fbxw7* mutation enhances T cell-involved immune responses, which is consistent with a previous transcription analysis.

### Drug screening demonstrates potential therapeutic strategies for ICC

FDA-approved drugs work only for a subset of tumor types. High-throughput drug screening was performed to explore novel drug treatment strategies. We utilized a drug library containing 144 compounds (Supplementary Table S4) for the preliminary screening of endogenous ICC tumors induced by HTV injection (Fig. 1A). ICC tumors were cultured as 3D organoids, and the third passage was used for drug testing. The half-maximal inhibitory concentration (IC50) was used to evaluate the drug sensitivity. Most drugs had little effect on all ICC organoids, and some showed distinct sensitivities among the different gene mutations (Supplementary Fig. S7D).

**Fig. 7:**
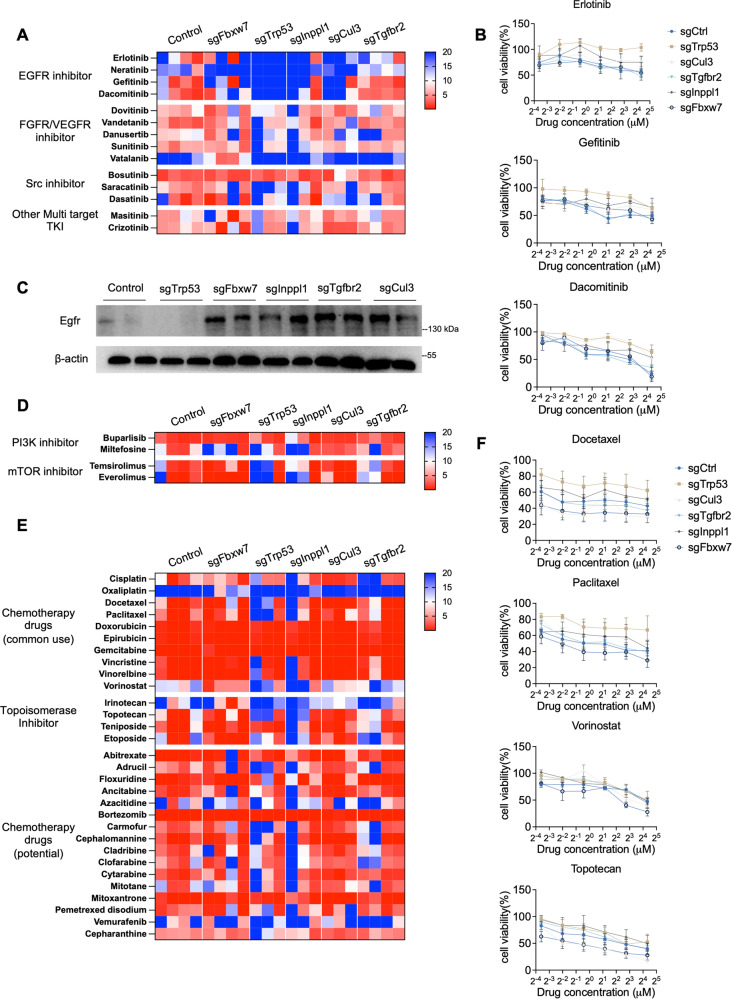
Drug screening reveals potential drug therapies in CRISPR-targeted gene-mutant ICC organoids. **A** Drug library screening of TKI inhibitors in 273cc organoids, which are derived from transplanted nude mice. **B** Drug response curves of EGFR inhibitors in mutant 273cc cell-developed organoids. **C** Western blot reveals Egfr expression in different gene-mutant 273cc cells. **D** Drug library screening of PI3K/mTOR inhibitors in 273cc organoids. **E** Drug library screening of chemotherapy drugs in 273cc organoids. **F** Drug response curves of representative chemotherapy drugs in mutant 273cc cell-developed organoids.

Next, we used mutant 273cc transplanted tumors to validate the effect of the sensitive drugs selected by preliminary screening. We examined different tyrosine kinase inhibitors (TKIs) except FDA-approved drugs for liver cancer, including EGFR inhibitors (erlorinib, neratinib, dacomitinib, and gefitinib), FGFR or VEGFR inhibitors (dovitinib, vandetanib, danusertib, sunitinib, and vatalanib), Src inhibitors (bosutinib, saracatinib, and dasatinib), and other multiple TKIs (masitinib and crizotinib). Most of the mutant organoids were sensitive to the aforementioned drugs, except for EGFR inhibitors (Fig. 7A). As Egfr expression was elevated in *Fbxw7*-, *Inppl1*-, *Tgfbr2*-, and *Cul3*-mutant cells, *Trp53*-mutant organoids were resistant to all Egfr inhibitors (Fig. 7B, C). Similar effects were observed in human cells (Supplementary Fig. S7G). The PI3K/AKT/mTOR pathway was activated in all mutant cells; thus, all mutant organoids were completely or partially sensitive to mTOR inhibitors (everolimus and temsirolimus) and a PI3K inhibitor (buparlisib) (Fig. 7D).

We also evaluated commonly used chemotherapy drugs and found that most worked efficiently, but some still had gene-specific responses. For example, we found that all organoids were sensitive to anthracyclines, including doxorubicin, epirubicin, and the antimetabolite drug gemcitabine. The taxane drugs paclitaxel and docetaxel had better efficiency in control, *Fbxw7*-, *Tgfbr2*- and *Cul3*-mutant organoids but not in *Trp53*- and *Inppl1*-mutant organoids. The histone deacetylase inhibitor vorinostat responded better to *Fbxw7*-mutant organoids. One type I topoisomerase inhibitor, topotecan, was most sensitive to *Fbxw7*- and *Cul3*-mutant organoids (Fig. 7E, F). Other drugs that are not typically used for treating liver cancer, but are approved for other types of tumors, also showed excellent responses to gene-mutant organoids (Fig. 7E). These drugs exhibit similar drug effects in human cells as well (Supplementary Fig. S7H, I, J).

Taken together, our results demonstrate distinct drug sensitivities among different gene-mutant tumors, and provide suggestions for novel drug therapies for ICC.

## Discussion

Our data provided a model of ICC in SPC mice. By introducing the CRISPR library into the mouse liver via HTV, we screened for genes that accelerated ICC formation within 4 months. We analyzed the signaling changes regulated by different gene mutations and provided potential drug therapy strategies for patients with ICC.

Our study identified different signaling pathways that are regulated in ICC tumors with different gene mutations. A previous study of genomic analysis in clinical samples identified the inflammation and proliferation classes in ICC [29, 30]. Our results showed that *Trp53*, *Fbxw7*, *Inppl1*, and *Tgfbr2* mutations enhanced ICC cell proliferation. The *Cul3* mutation appears to induce immune environment changes that contribute to tumor formation [9]. The proliferation class typically shows enrichment of classic oncogenic pathways, including RTK, VEGF, PDGF, RAS–MAPK, PI3K-AKT-mTOR, and WNT–β-catenin signaling pathways. Our study determined that the FGFR and VEGF pathways were upregulated. For the downstream pathways, because our mouse model contains a Pten mutation background, further gene mutations enhanced the PI3K signaling pathway, which may be due to the elevated signaling of Fgfr, Vegfr, or Igfr. These results indicate that PI3K is the predominant signaling pathway activated in ICC. The inflammation class exhibits enhanced immune response-related pathways. In *Fbxw7*-mutant tumors, many cytokines and chemokines were upregulated, and the key cytokine transducer, the Stat family, was also overexpressed. Moreover, *Fbxw7*-mutant tumors responded well to immune checkpoint inhibitors, indicating that Fbxw7 deficiency is likely to induce a severe immune response responsible for accelerating tumor formation and growth.

Our results demonstrate that targeting Trp53, Fbxw7, Inppl1, Tgfbr2, and Cul3 in hepatocytes using CRISPR/Cas9 generated ICC tumors in SPC mice, indicating five novel tumor suppressor genes for ICC. *Trp53* is a well-known tumor suppressor gene in many tumor types and has a high mutation rate in ICC. p53 inhibits cell division by binding to DNA and stimulating p21 expression. Fbxw7 is also a critical tumor suppressor in many cancers and functions as a substrate recognition component of the SCF E3 ubiquitin-protein ligase complex. Fbxw7 recognizes and binds to phosphorylated sites of oncoproteins, such as cyclin E, Myc, and Jun, and brings them to the SCF complex for ubiquitination and proteasomal degradation. Low expression of Fbxw7 was found in both HCC and ICC, and was associated with poor prognosis, advanced tumor stages, and metastasis [31, 32]. Inppl1, which encodes SH2-containing 5′-inositol phosphatase (SHIP2), is a member of the inositol 5-phosphatase family. Suppression of SHIP2 promotes tumorigenesis in gastric cancer cells, squamous cell carcinoma and thyroid carcinoma [23, 33, 34]. Here, we found that the downregulation of Inppl1 promoted PI3K signaling and enhanced cell proliferation, and that Inppl1 represents a new tumor suppressor gene in ICC. Tgfbr2 is one of the ligands of Tgf-β, and its binding induces phosphorylation of Tgfbr1, thus stimulating Smad signaling by phosphorylation of Smad2 and Smad3 and translocation to the nucleus along with Smad4 [35, 36]. Deletion or downregulation of Tgfbr2 is related to the progression of various types of tumors, including liver cancer. Here, we provide a new mouse model and evidence that Tgfbr2 downregulation promotes ICC formation, indicating that Tgfbr2 is a potent tumor suppressor of ICC. We also found that Cul3, a member of the largest E3 ubiquitin ligase family, facilitated ICC progression. However, unlike the other four genes, the *Cul3* mutation did not accelerate ICC cell proliferation but instead interfered with the tumor immune microenvironment. Cul3 deficiency promotes Areg secretion and induces chronic inflammation in the liver and CD8 T cell exhaustion by elevating PD1 expression [9]. Moreover, ICC tumors with low Cul3 expression are more sensitive to chemotherapy and targeted drugs, providing new therapeutic strategies for ICC tumors.

ICC lacks effective targeted therapies owing to intertumoral and intratumoral heterogeneities. The FDA has approved FGFR inhibitors and IDH inhibitors only for treating patients with FGFR2 fusion or IDH mutations, respectively [25–27]. The pan-FGFR inhibitor infigratinib worked effectively in most mutant ICC cells compared to the control and was more efficient than another FGFR inhibitor, pemigatinib, which exhibited better sensitivity only in *Tgfbr2-* and *Cul3*-mutant cells. Because most cells displayed elevated expression of Fgfr, Vegfr, and Igfr, multiple TKIs such as sorafenib, regorafenib, and lenvatinib, which are approved for treating HCC, could also be used for ICC therapy. Moreover, although lenvatinib showed a moderate response, it showed a synergistic effect when combined with pemigatinib and infigratinib. PI3K signaling was activated in all cells, and we found that the pan-PI3K inhibitor buparlisib efficiently affected ICC organoid growth, indicating that PI3K is a potential target for treating ICC tumors. We also found that most organoids were sensitive to the Src inhibitor bosutinib, which typically treats chronic myelogenous leukemia by inhibiting Bcr-Abl tyrosine kinase. Although bosutinib is a TKI, it also efficiently inhibits the PI3K-AKT-mTOR pathway [37], suggesting a novel therapy for ICC tumors.

Taken together, using an SPC mouse model, our results verified that *Trp53*, *Fbxw7*, *Inppl1*, *Tgfbr2,* and *Cul3* are tumor suppressor genes of ICC. However, the detailed mechanisms by which these genes initiate ICC formation and induce immune microenvironment changes need to be further elucidated. Here, we analyzed multiple signaling pathways regulated by these gene mutations and examined multiple drug responses, providing suggestions for novel therapeutic strategies for ICC tumors.

### Reporting summary

Further information on research design is available in the Nature Research Reporting Summary linked to this article.

### Supplementary information

Supplementary data

Supplemental file_Uncropped WB

Reporting Summary

## Data Availability

All raw RNA-seq data and processed read counts have been deposited in NCBI’s Gene Expression Omnibus (PRJNA955525). The data generated in this study are provided in the main article and Supplementary Data files.
