# Supplementary material for: The heterogeneity of signaling pathways and drug responses in intrahepatic cholangiocarcinoma with distinct genetic mutations
Source: Cell Death Dis. 2024 Jan 11;15(1):34. doi: 10.1038/s41419-023-06406-7 (PMC10784283; doi:10.1038/s41419-023-06406-7)
Supplement: Supplementary file 1 — Supplementary data [file 41419_2023_6406_MOESM1_ESM.pdf]

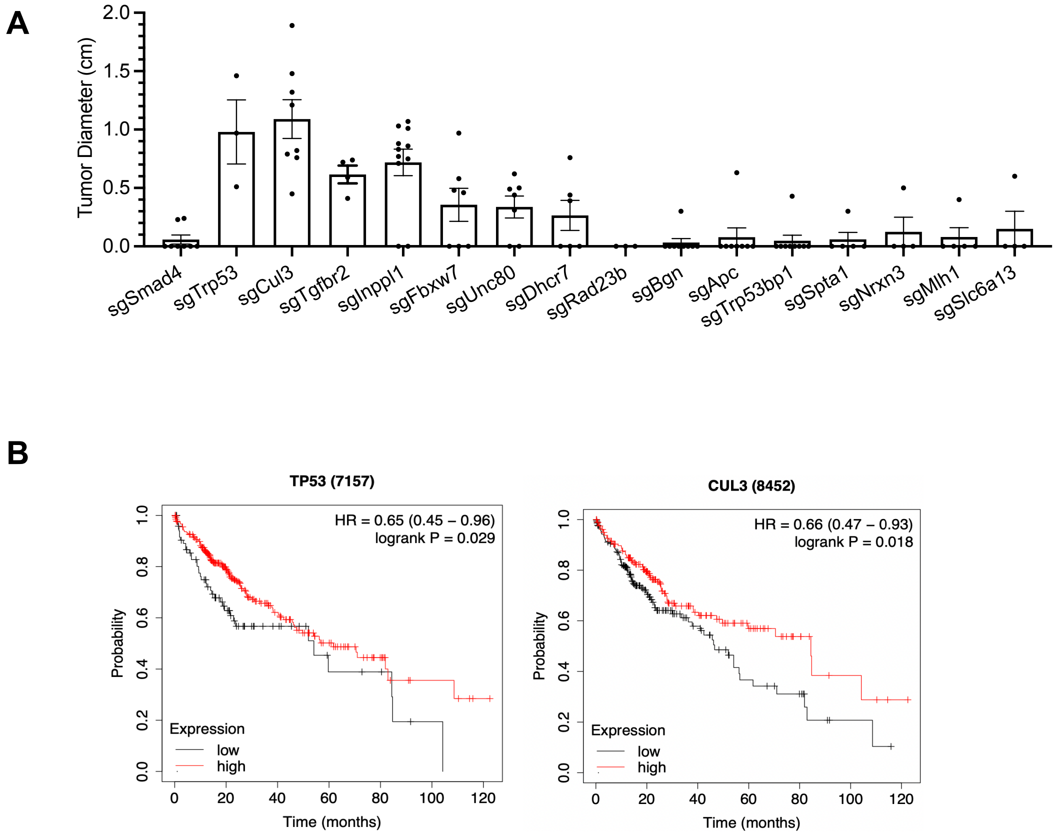

Supplementary Figure S1. The identified gene accelerates ICC formation in SPC mice.

(A) Diameter of the largest tumor collected from each mouse. sgSmad4 was included as a negative control. **(B)** Kaplan-Meier survival plots show survival probability with different gene expression of TP53 and CUL3.

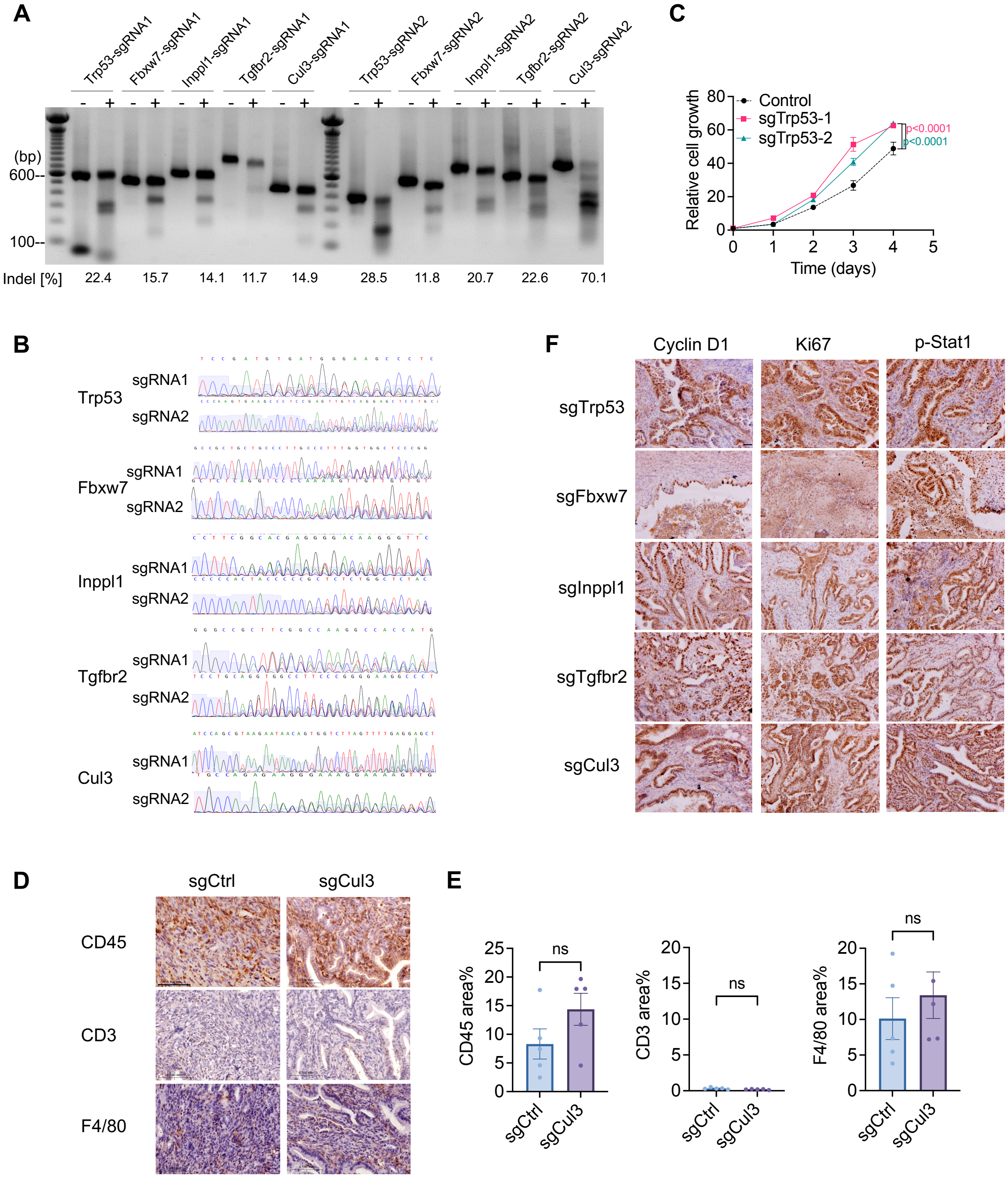

A
Trp53-sgRNA1
Fbxw7-sgRNA1
Inppl1-sgRNA1
Tgfbr2-sgRNA1
Cul3-sgRNA1
Trp53-sgRNA2
Fbxw7-sgRNA2
Inppl1-sgRNA2
Tgfbr2-sgRNA2
Cul3-sgRNA2
(bp)
600--
100--
Indel [%] 22.4 15.7 14.1 11.7 14.9 28.5 11.8 20.7 22.6 70.1
C
Control
sgTrp53-1
sgTrp53-2
p<0.0001
p<0.0001
Relative cell growth
Time (days)
B
Trp53 sgRNA1 sgRNA2
Fbxw7 sgRNA1 sgRNA2
Inppl1 sgRNA1 sgRNA2
Tgfbr2 sgRNA1 sgRNA2
Cul3 sgRNA1 sgRNA2
F
Cyclin D1
Ki67
p-Stat1
sgTrp53
sgFbxw7
sgInppl1
sgTgfbr2
sgCul3
D
sgCtrl
sgCul3
CD45
CD3
F4/80
E
CD45 area%
CD3 area%
F4/80 area%
ns
sgCtrl
sgCul3

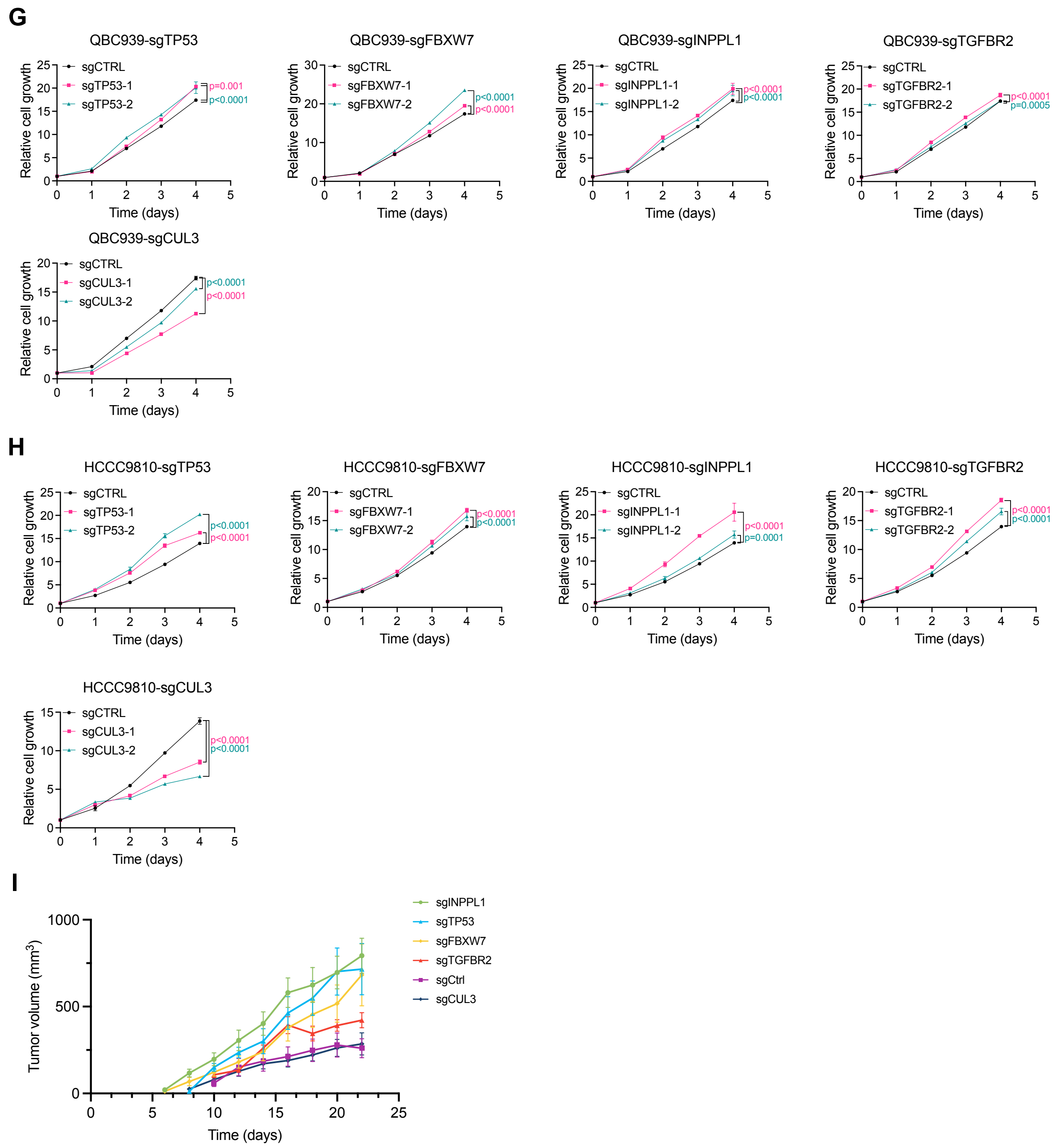

Supplementary Figure S2. The identified gene mutation accelerates cancer progression with different mechanisms. (A) Surveyor assay identified sgRNAs induced gene mutation in the targeted sequences. Indel frequencies are indicated. **(B)** Sanger sequencing indicate the mutated sequence context. **(C)** Cell proliferation assay in transfected 273cc cells. Trp53 gene sequences were modified by sgRNAs. Cell viability was examined by alamar blue, the fluorescence intensity was normalized to the values on day 0. **(D, E)** IHC staining and

statistic analysis of immune cells in ICC tumors with or without Cul3 mutation developed in Nude mice. CD45 indicates total immune cells; CD3 indicates T cells; F4/80 indicates macrophages. Scale bar=100 µm. **(F)** IHC staining of cell cycle and immune related molecules in ICC tumors developed in Nude mice. Scale bar=20 µm. ns: not significant. Cell proliferation assay in transfected QBC939 cells **(G)** and HCCC9810 cells **(H)**. Cell viability was examined by alamar blue, the fluorescence intensity was normalized to the values on day 0. **(I)** Tumor volume measured at different time points in nude mice bearing QBC939 cells transplanted with different sgRNAs (n=4 to 6 in each group).

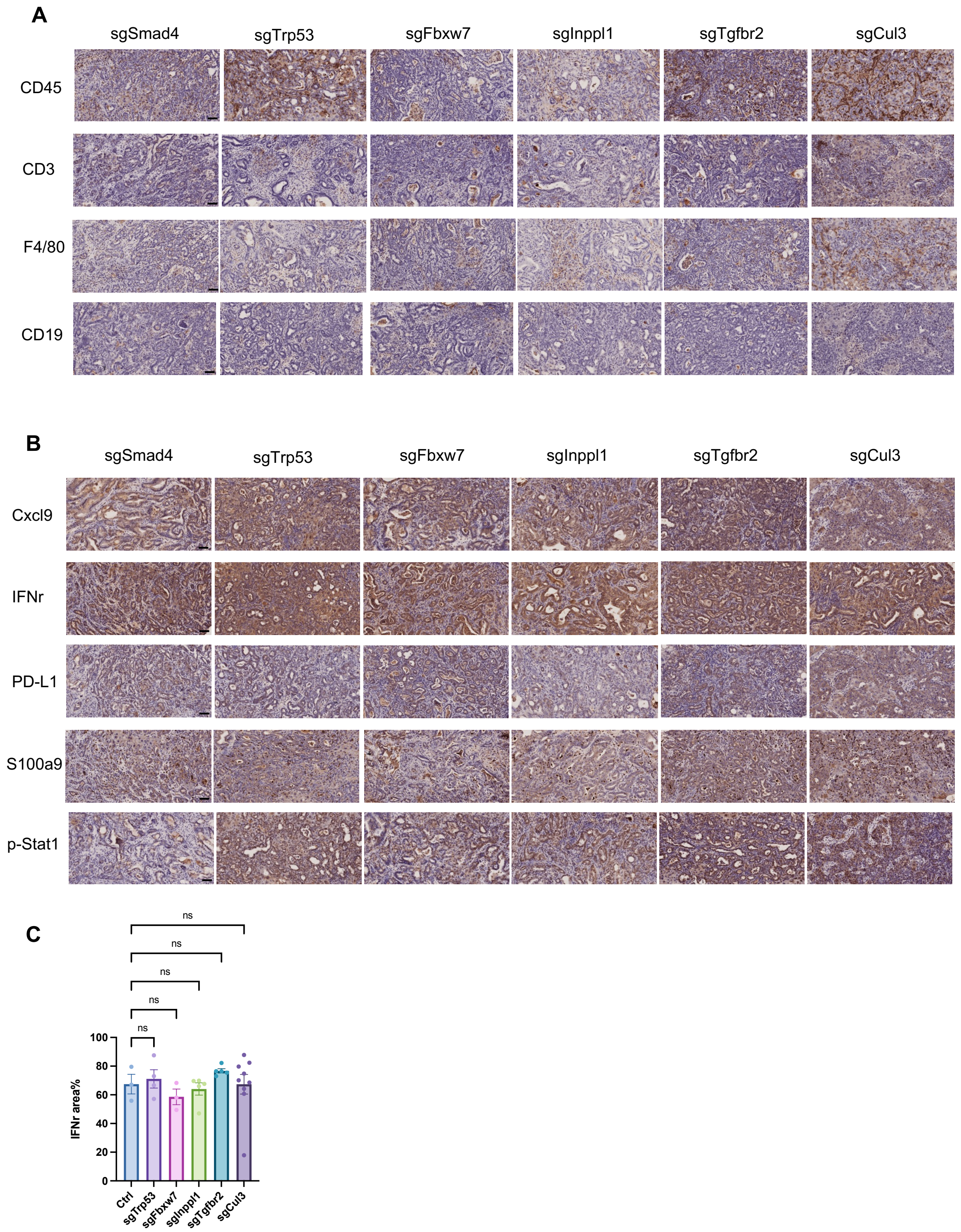

Supplementary Figure S3. Immune components vary in ICC tumors developed in SPC mice. (A) IHC staining of immune cells in ICC tumors. CD45 indicates total immune cells; CD3 indicates T cells; F4/80 indicates macrophages; CD19 indicates B cells. (B, C) IHC staining and statistical analysis of immune related molecules in ICC tumors. Scale bar=50 μm.

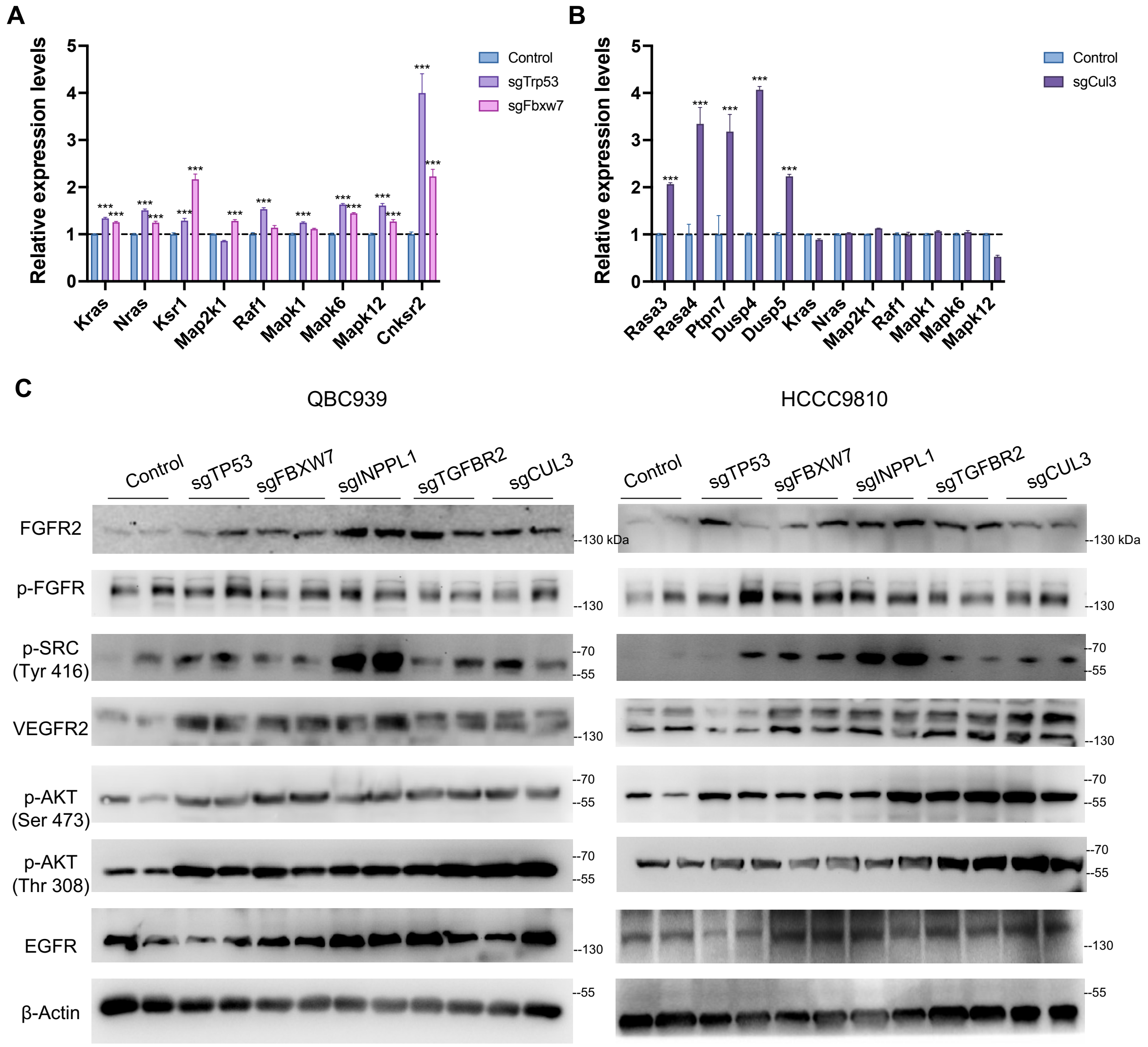

Supplementary Figure S4. RNA-seq reveal RAS-MAPK pathway changes in cells. (A) In Fbxw7 mutated cells, Kras and Nras, Ksr1, Map2k1 (Mek1), Mapk1 (Erl2) were activated, while in Trp53 mutated cells, Nras, Raf1, Cnksr2, Mapk12, Mapk6 were upregulated, indicating activation of MAPK pathway. **(B)** In Cul3 mutated cells, a group of RAS-MAPK inhibition related molecules were activated, such as Rasa3 and Rasa4 (negatively regulate RAS pathway), Ptpn7 (phosphate tyrosine-phosphorylated MAPK1), Dusp4 and Dusp5 (dephosphorylate both MAP kinases ERK1 and ERK2) were all upregulated, suggesting RAS-MAPK pathway is suppressed in Cul3 mutated cells. **(C)** Western blot revealed activation of VEGF, FGFR, EGFR and PI3K signaling of gene-mutant cells. Human cell line QBC939 and HCCC9810 were used.

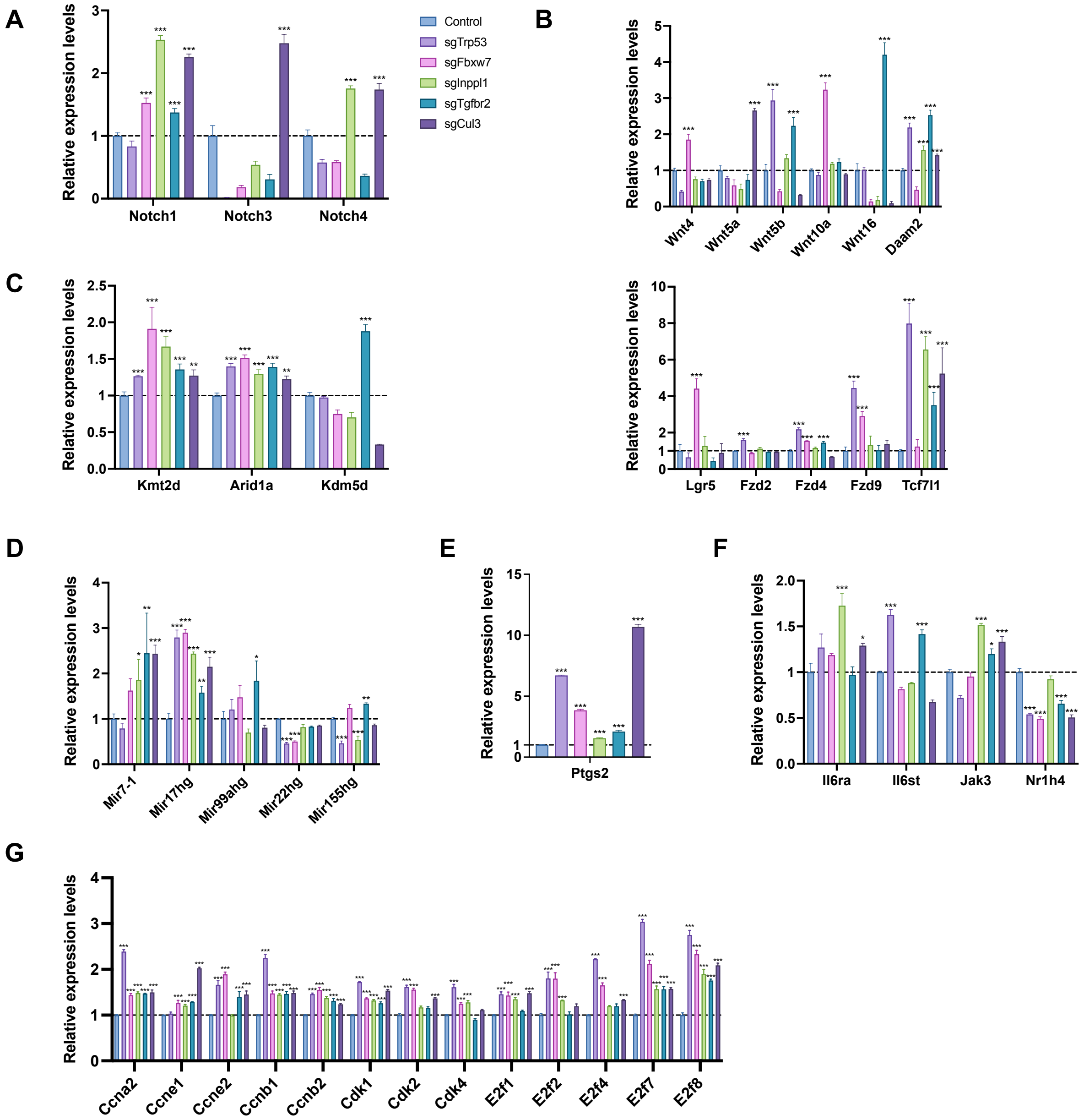

Supplementary Figure S5. RNA-seq reveal developmental pathway changes in cells.

Notch signaling (A), Wnt signaling (B), epigenetic dysregulation (C), non-coding RNAs (ncRNAs) (D), chronic inflammation (E, F), cell cycle and cell proliferation (G) pathway related molecules are affected after gene mutation.

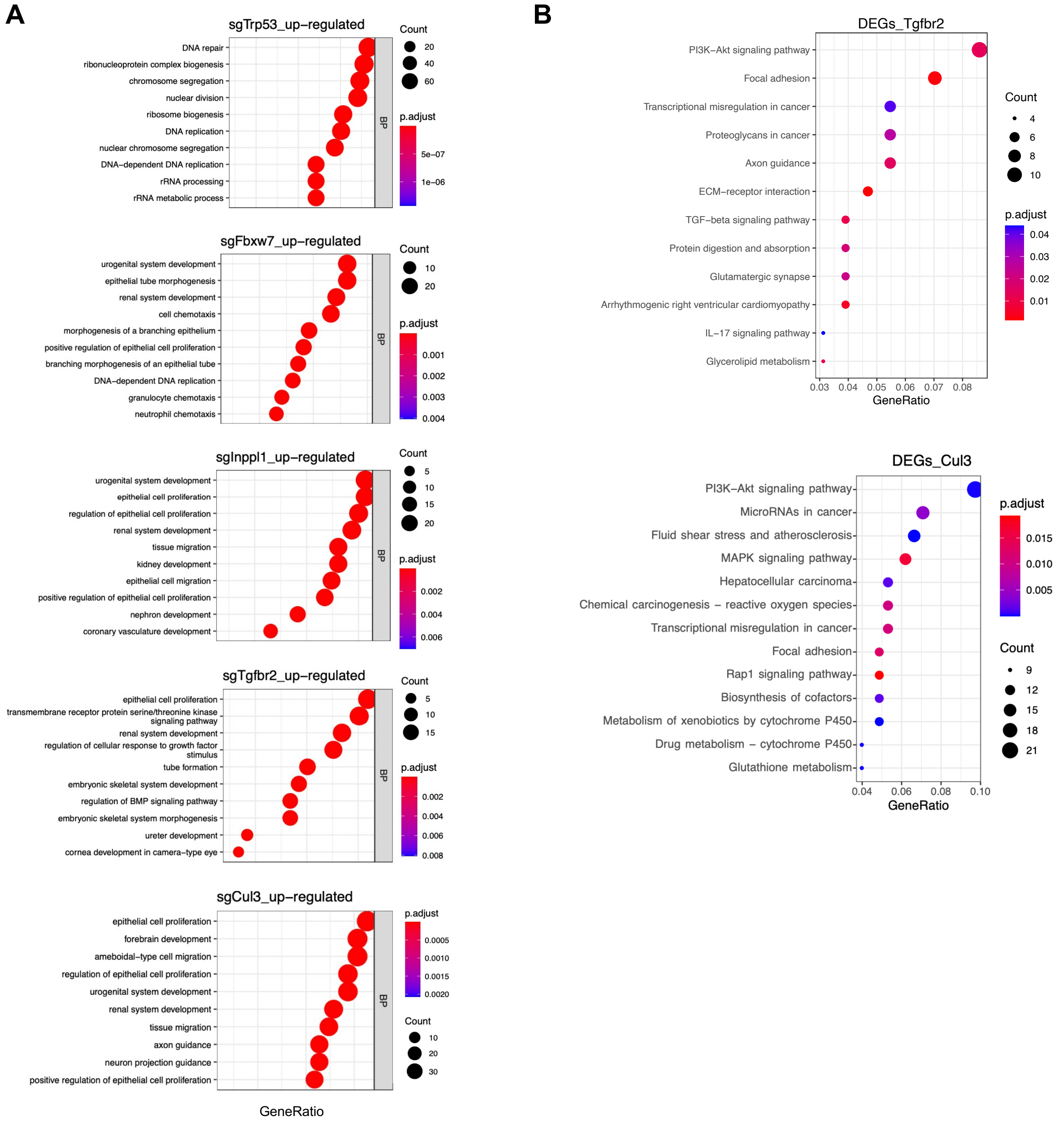

Supplementary Figure S6. GO and KEGG enrichment of RNA-seq data in gene mutated 273cc cells. (A) GO enrichment analysis of all the five mutated genes reveal upregulated pathways. Count and p.adjust value were indicated. (B) Top upregulated KEGG pathways were showed in *Tgfbr2* and *Cul3*-mutant cells.

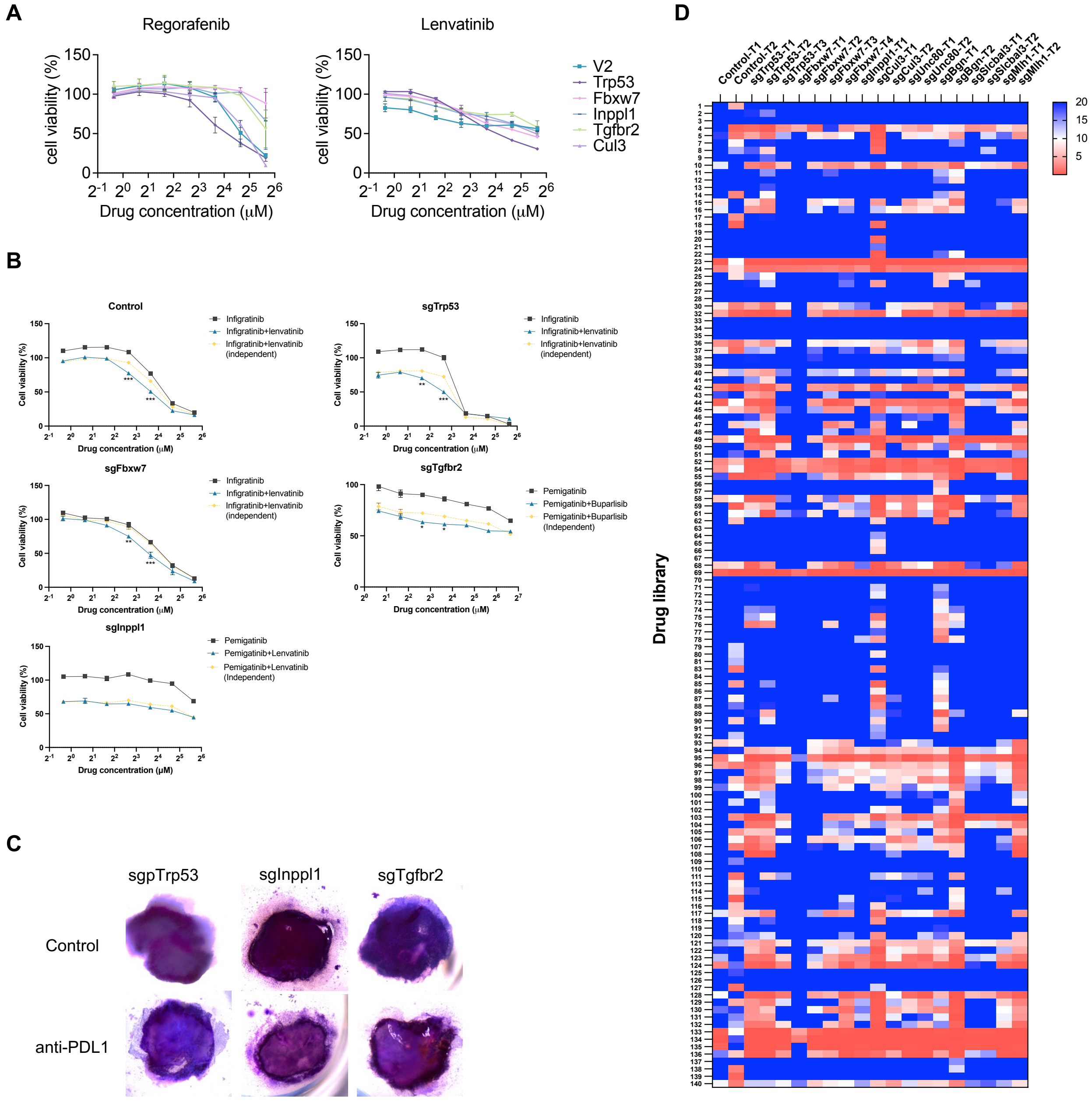

A
Regorafenib
Lenvatinib
cell viability (%)
Drug concentration (μM)
V2
Trp53
Fbxw7
Inppl1
Tgfbr2
Cul3
B
Control
sgTrp53
sgFbxw7
sgTgfbr2
sgInppl1
Cell viability (%)
Drug concentration (μM)
Infigratinib
Infigratinib+lenvatinib
Infigratinib+lenvatinib (independent)
Pemigatinib
Pemigatinib+Buparlisib
Pemigatinib+Buparlisib (Independent)
Pemigatinib+Lenvatinib
Pemigatinib+Lenvatinib (Independent)
C
sgpTrp53
sgInppl1
sgTgfbr2
Control
anti-PDL1
D
Control-T1
Control-T2
sgTrp53-T1
sgTrp53-T2
sgTrp53-T3
sgFbxw7-T1
sgFbxw7-T2
sgFbxw7-T3
sgFbxw7-T4
sgInppl1-T1
sgCul3-T1
sgCul3-T2
sgUnc80-T1
sgUnc80-T2
sgBgn-T1
sgBgn-T2
sgSlcbal3-T1
sgSlcbal3-T2
sgMlh1-T1
sgMlh1-T2
Drug library
20
15
10
5

E

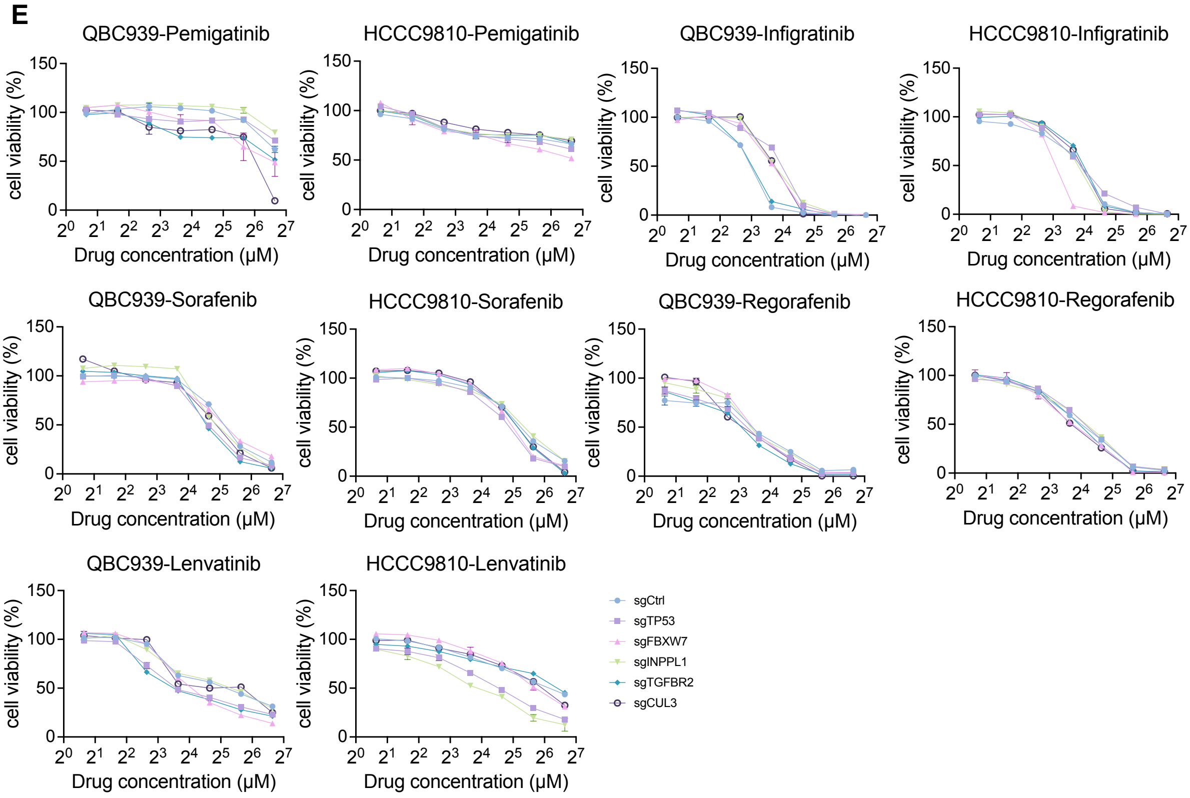
QBC939-Pemigatinib
HCCC9810-Pemigatinib
QBC939-Infigratinib
HCCC9810-Infigratinib
QBC939-Sorafenib
HCCC9810-Sorafenib
QBC939-Regorafenib
HCCC9810-Regorafenib
QBC939-Lenvatinib
HCCC9810-Lenvatinib
cell viability (%)
Drug concentration (μM)
sgCtrl
sgTP53
sgFBXW7
sgINPPL1
sgTGFBR2
sgCUL3

F

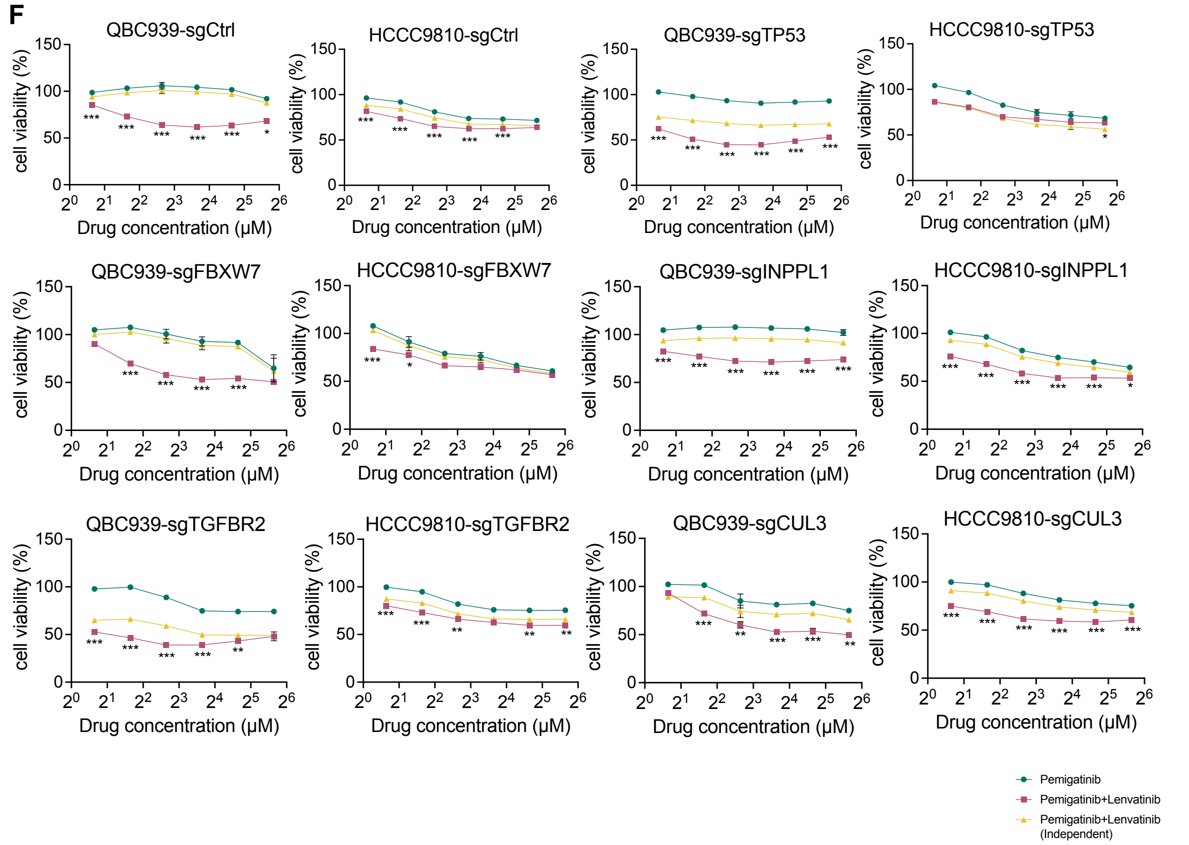
QBC939-sgCtrl
HCCC9810-sgCtrl
QBC939-sgTP53
HCCC9810-sgTP53
QBC939-sgFBXW7
HCCC9810-sgFBXW7
QBC939-sgINPPL1
HCCC9810-sgINPPL1
QBC939-sgTGFBR2
HCCC9810-sgTGFBR2
QBC939-sgCUL3
HCCC9810-sgCUL3
cell viability (%)
Drug concentration (μM)
Pemigatinib
Pemigatinib+Lenvatinib
Pemigatinib+Lenvatinib
(Independent)

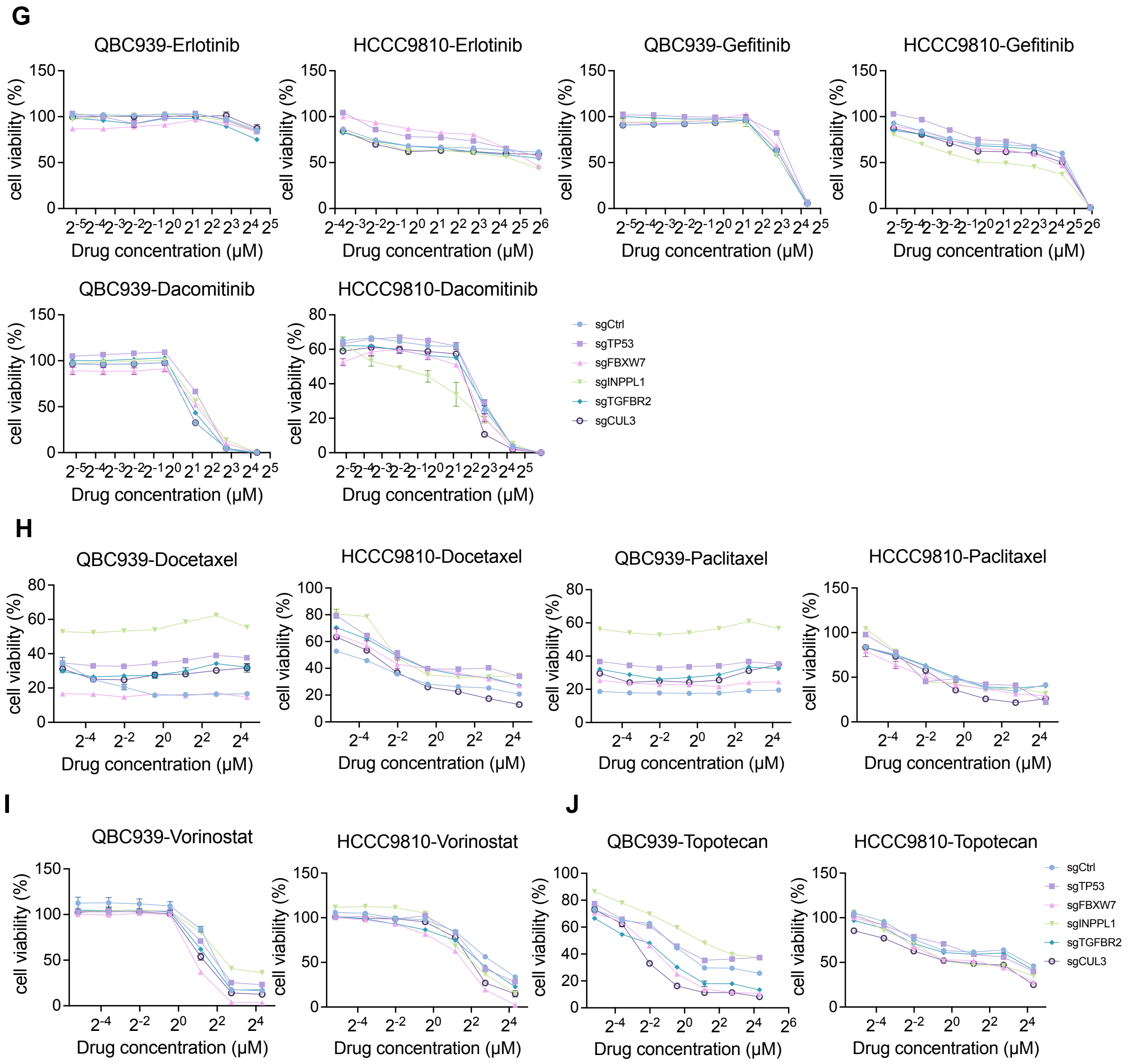

Supplementary Figure S7. Drug effect of mutated 3D organoids and cells. (A) Drug response curves of different drugs in gene mutated 273cc cells. **(B)** Drug combination treatment on gene mutated 273cc cells. Drug concentrations for pemigatinib and Infigrabtinib range from 50 µM to 0.78 µM (2-fold dilution) for most of cells. For Tgfbr2 mutated cells, pemigatinib range from 100 µM to 1.56 µM. 5 µM of Lenvatinib or 1 µM of Buparlisib was used for drug combination. Two-way ANOVA was used for statistical analysis. Independent combination curves were calculated by using Bliss model. **(C)** MTT staining of gene mutated 3D-TSCs after anti-PDL1 treatment. **(D)** Drug screening of different gene mutated 3D organoids developed in SPC mice. 140 drugs were used for screening. Drug response curves of TKI **(E)**, EGFR inhibitors **(G)**, taxane drugs **(H)**, histone deacetylase inhibitor Vorinostat

(I) and type I topoisomerase inhibitor Topotecan **(J)** in gene mutated QBC939 and HCCC9810 cells. **(F)** Drug combination treatment on gene mutated QBC939 and HCCC9810 cells. Drug concentrations for pemigatinib range from 50 μM to 1.56 μM (2-fold dilution) for most of cells. Lenvatinib (6.25 μM) was used for drug combination. Two-way ANOVA was used for statistical analysis. Independent combination curves were calculated by using Bliss model. *p<0.05, **p<0.01, ***p<0.001.

Supplementary Table S1. Gene mutation frequencies in human ICC

Zhu AX, et al. (2014)(1)		Valle JW, et al. (2017)(2)		Javle M, et al. (2016)(3)		Chong DQ and Zhu AX (2016)(4)		Ross JS, et al. (2014)(5)	
BRAF	4.9%	TP53	2.5%-44.4%	TP53	27%	KRAS	9%-24%	ARID1A	36%
CTNNB1	0.6%	IDH1/2	4.9%-36%	KRAS	22%	PIK3CA	4%	IDH1/2	36%
KRAS	8.6%	ARID1A	6.9%-36%	PI3KCA	5%	BRAF	5%	TP53	36%
MAP2K1	1.9%	CDKN2A/B	5.6%-25.9%	BAP1	15%	IDH1/IDH2	16%-36%	FGFR2	14%
NRAS	3.1%	KRAS	8.6%-24.2%	CDKN2A/B	27%	TP53	3%-36%	KRAS	11%
PIK3CA	4.3%	SMAD4	3.9%-16.7%	ARID1A	18%	ARID1A	19%-36%	PTEN	11%
PTEN	0.6%	MLL3	14.8%	BRAF	5%	PTEN	1%-11%	CDKN2A	7%
TP53	2.5%	BAP1	13.0%	IDH1/2	20%	PBRM1	11%-17%	CDK6	7%
IDH1	15.5%	PTEN	0.6%-11%			BAP1	9%-25%	ERBB3	7%
IDH2	4.5%	ARAF	11.0%			SMAD4	4%	MET	7%
		RNF43	9.3%			FBXW7	6%	NRAS	7%
		ROBO2	9.3%			CDKN2A	7%	BRCA1	4%
		GNAS	9.3%			CDK6	7%	BRCA2	4%
		PIK3CA	3%-9%			BRCA	4%	NF1	4%
		BRAF	3%-7.1%			NF1	4%	PIK3CA	4%
		ERBB3	7%			TSC1	4%	PTCH1	4%
		NRAS	1.5%-7%					TSC1	4%
		CDK6	7%						
		ERBB3	7.0%						
		PEG3	5.6%						
		XIRP	5.6%						
		RB1	5.0%						
		MET	4.7%						
		BRCA1/2	4.0%						
		NF1	4.0%						
		TSC1	4.0%						
		RADIL	3.7%						
		NDC80	3.7%						
		PCDHA13	3.7%						
		LAMA2	3.7%						
		EGFR	1.5%-2%						
		CTNNB1	0.6%						

Simbolo M.et al (2014)(6)		Ettrich TJ, et al. (2019)(7)		Carotenuto M, et al. (2022)(8)		Tian W, et al. (2020)(9)		Zheng B, et al. (2023)(10)		Sara EY, et al. (2023)(11)	
AKT1	2.8%	TP53	22%	IDH1/2	10–20%	TP53	56.82%	KRAS	44.2%	TP53	20-42%
APC	1.4%	ARID1A	9%	FGFR	7–16%	KRAS	29.55%	TP53	37.2%	FGFR2	10-20%
ARID1A	11.4%	KRAS	22%	BRAF V600E	5%	SMAD4	18.18%	ARID1A	18.6%	IDH1/2	10-15%
BAP1	14.3%	IDH1	4%	KRAS	8–54%	TERT	20.45%	SMAD4	18.6%	ARID1A	11-22%
BRAF	4.3%	BAP1	9%	ERBB2	8%	ARID1A	13.64%	CDKN2A	14.0%	CDKN2A/B	2-30%
CDKN2A	1.4%	PBRM1	9%	PIK3CA	7%	CDKN2A	18.18%	BRCA2	11.6%	KRAS	8-25%
ERBB4	1.4%	SMAD4	9%	NTRK	≤ 1%	STK11	6.82%	VEGFA	11.6%	BAP1	15-25%
FBXW7	1.4%	PIK3CA	9%	BRCA1	0.40%	CCND1	9.09%	KEAP1	9.3%	SMAD4	5-10%
FGFR3	2.8%	FBXW7	4%	BRCA2	2.70%	FGF19	9.09%	MYC	9.3%	BRAF	2-7%
IDH1	15.7%			TP53	20%	FGF3	9.09%	STK11	9.3%	ERBB	1-8%
IDH2	4.3%			AXIN1	41%	FGF4	9.09%			BRCA1	1%
KDR	1.4%			APC	13%	FGFR2	9.09%			BRCA2	2.40%
KRAS	15.7%			CDH1	11%	PBRM1	9.09%			NTRK	1-3.5%
NRAS	9.3%			CTNNB1	8%	FAT3	6.82%			EGFR	0-8%
PBRM1	14.3%					FRS2	6.82%			HER2	1-6%
PIK3CA	5.7%					GLI3	6.82%			MSI-H	1-2%
PIK3C2A	7.1%					GNAS	6.82%			MDM2	<4%
PIK3C2G	4.3%					KMT2C	6.82%			TERT	6%
PTEN	1.4%					MAGI2	6.82%				
SMAD4	1.4%					MDM2	6.82%				
STK11	1.4%					MET	6.82%				
TGFBR2	4.3%					NF1	6.82%				
TP53	8.6%					TGFBR2	6.82%				
						APC	4.55%				
						FBXW7	4.55%				
						KEAP1	4.55%				
						PIK3CA	4.55%				
						PTEN	4.55%				
						SMARCA4	4.55%				
						BRCA1	4.55%				
						BRCA2	4.55%				
						CUL3	2.27%				

Supplementary Table S2. **Pathways upregulated in most mutated cells.**

Upregulated pathways		Pathway-related genes	Mutated genes
**Receptor tyrosine kinase (RTK)**	VEGF	Vegfd, Kdr, Flt1	Trp53, Fbxw7, Inppl1, Tgfbr2, Cul3
		Nrp2	Fbxw7
	FGF	Fgfr1	Trp53, Fbxw7, Inppl1, Cul3
	IGF	Igfbp3, Irs2	Cul3
		Igfbp3, Irs1, Irs2	Fbxw7
		Igfbp5, Igfbp2	Inppl1
		Igfbp5, Igfbp2, Irs2	Tgfbr2
		Igfbp2, Igfbp3, Igfbp5, Irs1, Irs2	Trp53
**PI3K/mTOR**		Pik3r5	Trp53, Inppl1, Tgfbr2, Cul3
		Prr5l	Tgfbr2
**Notch**		Notch1	Cul3, Fbxw7, Inppl1
		Notch3	Cul3
		Notch4	Inppl1
**Wnt/β-catenin**		Wnt5a, Icf7l1	Cul3
		Wnt4, Wnt10a, Fzd9, Lgf5	Fbxw7
		Daam2, Wnt5b, Wnt16, Icf7l1	Tgfbr2
		Fzd2, Fzd4, Fzd9, Daam2, Wnt5b	Trp53
**Inflammation**		Ptgs2	Trp53, Fbxw7, Inppl1, Tgfbr2, Cul3
	IL-6	Il6ra	Fbxw7, Inppl1, Cul3
		Il6st	Trp53, Tgfbr2
	JAK/STAT	Jak3	Inppl1, Tgfbr2, Cul3
		Jak1	Trp53, Fbxw7
		Stat2	Fbxw7
		Nr1h4 downregulated	Trp53, Fbxw7, Tgfbr2, Cul3
**Cell cycle**		Ccna2 (Cyclin A), Ccne2 (Cyclin E), Ccnb1 (Cyclin B), Cdk1, Cdk2, Cdk4, E2f1, E2f2, E2f4, E2f7, E2f8	Trp53
		Ccne2, Ccnb2, Cdk2, E2f1, E2f2, E2f4, E2f7, E2f8	Fbxw7

	Ccna2, Ccnb1, Ccnb2, E2f1, E2f7, E2f8	Inppl1
	Ccna2, Ccne2, Ccnb1, Ccnb2, E2f7, E2f8	Tgfbr2
	Ccna2, Ccne1, Cdk1, E2f1, E2f7, E2f8	Cul3

**Epigenetic process**	Upregulated		Kmt2d, Arid1a	Trp53, Fbxw7, Inppl1, Tgfbr2, Cul3
			Kdm5d	Tgfbr2
		Noncoding RNAs (ncRNAs)	Mir17hg	Cul3, Fbxw7, Inppl1, Tgfbr2, Trp53
			Mir7-1	Cul3, Fbxw7, Inppl1, Tgfbr2
			Mir155hg	Tgfbr2, Trp53
			Mir99ahg	Tgfbr2
			Mir22hg	Trp53
	Downregulated		Mir22hg	Fbxw7
			Mir155hg	Inppl1

Supplementary Table S3. **Distinct pathways upregulated after different gene mutations**.

<table>
<tr><th>Mutated gene</th><th colspan="2">Upregulated pathways</th><th>Pathway-related genes</th></tr>
<tr><td rowspan="3">Trp53</td><td colspan="2">Ribosome biogenesis</td><td>Nop56, Nop58</td></tr>
<tr><td rowspan="2">Nucleocytoplasmic transport</td><td>Nuclear pore complex</td><td>Ndc1, Nup37, Nup43, Nup85, Nup107, Nup160, Nup205, Nup210, Sumo2</td></tr>
<tr><td>Nuclear transport complex</td><td>Ipo5, Xpo1, Xpo4, Cse1l</td></tr>
<tr><td rowspan="5">Fbxw7</td><td rowspan="5">Cytokine–cytokine receptor interaction</td><td>Chemokines</td><td>Ccl9, Cxcl3, Ppbp (Cxcl7), Ackr3 (Cxcr7), Cxcl10, Cxcl11, Cxcl14, Cxcl17</td></tr>
<tr><td>Interleukins</td><td>Il11, Il24</td></tr>
<tr><td>Interleukin receptors</td><td>Il1rn, Il1rl1, Il22ra1, Il12rb1, Il17re</td></tr>
<tr><td>Colony-stimulating factors</td><td>Csf3</td></tr>
<tr><td>Tumor necrosis factor (TNF) ligand family</td><td>Tnfsf9</td></tr>
<tr><td>Inppl1</td><td colspan="2">Apelin signaling</td><td>Apln, Apela</td></tr>
<tr><td>Tgfbr2</td><td colspan="2">Mitotic spindle</td><td>Bub1, Bub1b, Mad2l1, Cdc20, Cenpe</td></tr>
<tr><td rowspan="2">Cul3</td><td colspan="2">Reactive oxygen species and Cytochrome P450</td><td>Gsto1, Gsta1, Gsta2, Gsto2, Hmox1, Prkd1, Mgst2, Nqo1, Cyp1a1</td></tr>
<tr><td colspan="2">Polo-like kinase (PLK) and Aurora kinase</td><td>Plk1, Plk3, Aurka, Aurkb</td></tr>
</table>

Supplementary Table S4. The drug library containing 144 drugs.

No.	Brand	Item
1	Selleckchem	10-DAB (10-Deacetylbaccatin)
2	Selleckchem	2-Methoxyestradiol
3	Selleckchem	Abiraterone acetate
4	Selleckchem	Abitrexate (Methotrexate)
5	Selleckchem	Adrucil (Fluorouracil)
6	Selleckchem	Afatinib (BIBW2992)
7	Selleckchem	Altretamine (Hexalen)
8	Selleckchem	Amuvatinib (MP-470)
9	Selleckchem	Anastrozole (Arimidex)
10	Sigma	Ancitabine hydrochloride
11	Selleckchem	Artemether (SM-224)
12	Selleckchem	Aspirin (Acetylsalicylic acid)
13	Selleckchem	Atazanavir sulfate
14	Selleckchem	Axitinib
15	Selleckchem	Azacitidine (Vidaza)
16	Selleckchem	Azaguanine-8
17	Selleckchem	Azithromycin (Zithromax)
18	Selleckchem	Bendamustine hydrochloride
19	Selleckchem	Bepotastine Besilate
20	Selleckchem	Bergapten
21	Selleckchem	Bindarit
22	Selleckchem	Bleomycin sulfate
23	BOC Sciences	Bortezomib
24	Selleckchem	Bosutinib (SKI-606)
25	Selleckchem	Busulfan
26	Sigma	Cantharidin
27	Selleckchem	Capecitabine (Xeloda)
28	Selleckchem	Carbazochrome sodium sulfonate
29	Selleckchem	Carboplatin (Diamine(1,1-cyclobutane-dicarboxylato)platinum(II))
30	Selleckchem	Carmofur
31	Sigma	Carmustine
32	Selleckchem	Cephalomannine
33	Selleckchem	Chlorambucil
34	Selleckchem	Cinacalcet hydrochloride (AMG-073 HCl)
35	Sigma	Cisplatin (cis-Diammineplatinum(II) dichloride)

36	Selleckchem	Cladribine
37	Selleckchem	Clofarabine
38	Selleckchem	Clomifene citrate (Serophene)
39	Selleckchem	Clorsulon
40	Selleckchem	Crizotinib (PF-02341066)
41	Selleckchem	Cyclophosphamide monohydrate
42	Selleckchem	Cytarabine
43	Selleckchem	Dacarbazine (DTIC-Dome)
44	Selleckchem	Dacomitinib (PF299804,PF-00299804)
45	Selleckchem	Danusertib (PHA-739358)
46	Selleckchem	DAPT (GSI-IX)
47	J&K	Dasatinib
48	Selleckchem	Diethylstilbestrol (Stilbestrol)
49	Selleckchem	Docetaxel (Taxotere)
50	Selleckchem	Dovitinib (TKI-258)
51	Selleckchem	DOXIFLURIDINE
52	Selleckchem	Doxorubicin (Adriamycin)
53	Selleckchem	Eltrombopag (SB-497115-GR)
54	J&K	Epirubicin hydrochloride
55	Selleckchem	Erlotinib hydrochloride
56	Selleckchem	Esomeprazole sodium (Nexium)
57	Sigma	Estramustine
58	Selleckchem	Etoposide (VP-16)
59	Selleckchem	Everolimus (RAD001)
60	Selleckchem	Famciclovir (Famvir)
61	Selleckchem	Floxuridine
62	Selleckchem	Fludarabine (Fludara)
63	Selleckchem	Flunarizine dihydrochloride
64	Selleckchem	Flutamide (Eulexin)
65	Selleckchem	Formestane
66	Selleckchem	Ftorafur (Tegafur)
67	Selleckchem	Fulvestrant (Faslodex)
68	Selleckchem	Gefitinib (Iressa)
69	Selleckchem	Gemcitabine Hydrochloride
70	Selleckchem	Geniposidic acid
71	Selleckchem	Genistein
72	Selleckchem	Histamine dihydrochloride
73	Selleckchem	Ifosfamide

74	Selleckchem	Imatinib Mesylate
75	Selleckchem	Irinotecan
76	Selleckchem	Itraconazole (Sporanox)
77	Selleckchem	Lamotrigine
78	Selleckchem	Lapatinib Ditosylate (Tykerb)
79	Selleckchem	Lenalidomide
80	Selleckchem	Letrozole
81	Selleckchem	Linagliptin (BI-1356)
82	Selleckchem	Lincomycin hydrochloride (Lincocin)
83	Selleckchem	Lomustine (CeeNU)
84	Selleckchem	Lonidamine
85	Selleckchem	Masitinib
86	Selleckchem	MDV3100 (Enzalutamide)
87	Selleckchem	Mechlorethamine HCL
88	Selleckchem	Megestrol acetate
89	Selleckchem	Mercaptopurine
90	Selleckchem	Mesna (Uromitexan, Mesnex)
91	Selleckchem	Methacycline hydrochloride (Physiomycine)
92	Selleckchem	Methazolastone (Temozolomide)
93	Selleckchem	Miltefosine (hexadecyl phosphocholine)
94	Selleckchem	Mitotane (Lysodren)
95	Selleckchem	Mitoxantrone
96	Selleckchem	Moroxydine
97	Selleckchem	Naloxone HCl
98	Selleckchem	Nelarabine (Arranon)
99	Selleckchem	Neratinib
100	Selleckchem	Nilotinib (AMN-107)
101	Selleckchem	Nilvadipine (ARC029)
102	Selleckchem	Oxaliplatin (Eloxatin)
103	Selleckchem	Paclitaxel (Taxol)
104	Selleckchem	Paeoniflorin
105	Selleckchem	Pazopanib hydrochloride
106	Selleckchem	Pemetrexed disodium
107	Selleckchem	Phenylbutazone (Butazolidin, Butatron)
108	Selleckchem	Phenylbutyric acid (Chlorambucil)
109	Selleckchem	Pioglitazone (Actos)
110	Selleckchem	Pomalidomide
111	Selleckchem	Ponatinib (AP24534)

112	Sigma	Proadifen hydrochloride
113	Selleckchem	Procarbazine hydrochloride
114	Selleckchem	Regorafenib (BAY 73-4506)
115	Selleckchem	Rosiglitazone (Avandia)
116	Selleckchem	Ruxolitinib (INCB018424)
117	Selleckchem	Saracatinib
118	Selleckchem	Sorafenib (Nexavar)
119	Selleckchem	Streptozotocin (Zanosar)
120	Selleckchem	Sulindac (Clinoril)
121	Selleckchem	Sunitinib Malate (Sutent)
122	Selleckchem	Temocapril hydrochloride
123	Selleckchem	Temsirolimus (Torisel)
124	J&K	Teniposide
125	Selleckchem	Tofacitinib citrate (CP-690550 citrate)
126	Selleckchem	Tolbutamide
127	Selleckchem	Tolnaftate
128	Selleckchem	Topotecan hydrochloride
129	Selleckchem	Tretinoin (Aberela)
130	Selleckchem	Vandetanib (Zactima)
131	Selleckchem	Vatalanib
132	Selleckchem	Vemurafenib (PLX4032)
133	Selleckchem	Vincristine
134	Selleckchem	Vinorelbine (Navelbine)
135	Selleckchem	Vismodegib (GDC-0449)
136	Selleckchem	Vorinostat (SAHA)
137	Selleckchem	XL-184 (Cabozantinib)
138	Selleckchem	Zoledronic acid
139	LKT	Toremifene
140	Selleckchem	Cepharanthine
141	Selleckchem	Lenvatinib
142	Selleckchem	Pemigatinib
143	Selleckchem	Infigratinib (BGJ398)
144	Selleckchem	Buparlisib (BKM120)

Supplementary Table S5. Primers for amplifying sgRNA targeted sequences

Gene		Forward	Reverse
Trp53	sgRNA1	ACACCTGATCGTTACTCGGC	AATTACAGACCTCGGGTGGC
	sgRNA2	TGGTGTTGGGCTGGTAGGCTGA	AGGAAGCCCAGGTGGAAGCCAT
Fbxw7	sgRNA1	TCCACTCTTACACCTAGGCTCA	CAAGCAAACGAGCGAAGCAT
	sgRNA2		
Inppl1	sgRNA1	GGGACCCCCATCTCAAATCA	TAGGGGAGACAGTGGGTTCA
	sgRNA2	TCCCTTCCTTGCCAGTTCTT	ACCCTACTCTACCCAGCACA
Tgfbr2	sgRNA1	GGGGATTGCCATAGCTGTCA	GCTGATGACATGCCTCGTGA
	sgRNA2	TCCACGTGCGCCAACAACATCA	GCCCGAAGTCACACAGGCAACA
Cul3	sgRNA1	TGTTCTCCCTTTTCTCTCTTGG	GCTTCATGTAGCCTTGAAAAGAA
	sgRNA2	ACTTGAGAGGTACTAGAAATTTCCA	GAGAGCTCTTGGAGGGGAAAAA

Supplementary Table S6. sgRNAs for single gene knockout in human cells (From Human CRISPR Knockout Pooled Library # 1000000048 (12)).

TP53	HGLibA_51047, HGLibA_51048
FBXW7	HGLibA_17153, HGLibA_17154
INPPL1	HGLibA_23363, HGLibA_23364
TGFBR2	HGLibA_49225, HGLibA_49226
CUL3	HGLibA_11778, HGLibA_11779

Reference

1. Zhu AX, Borger DR, Kim Y, Cosgrove D, Ejaz A, Alexandrescu S, et al. Genomic profiling of intrahepatic cholangiocarcinoma: refining prognosis and identifying therapeutic targets. Ann Surg Oncol. 2014;21(12):3827-34.
2. Valle JW, Lamarca A, Goyal L, Barriuso J, Zhu AX. New Horizons for Precision Medicine in Biliary Tract Cancers. Cancer Discov. 2017;7(9):943-62.
3. Javle M, Bekaii-Saab T, Jain A, Wang Y, Kelley RK, Wang K, et al. Biliary cancer: Utility of next-generation sequencing for clinical management. Cancer. 2016;122(24):3838-47.
4. Chong DQ, Zhu AX. The landscape of targeted therapies for cholangiocarcinoma: current status and emerging targets. Oncotarget. 2016;7(29):46750-67.
5. Ross JS, Wang K, Gay L, Al-Rohil R, Rand JV, Jones DM, et al. New routes to targeted therapy of intrahepatic cholangiocarcinomas revealed by next-generation sequencing. Oncologist. 2014;19(3):235-42.
6. Simbolo M, Fassan M, Ruzzenente A, Mafficini A, Wood LD, Corbo V, et al. Multigene mutational profiling of cholangiocarcinomas identifies actionable molecular subgroups. Oncotarget. 2014;5(9):2839-52.
7. Ettrich TJ, Schwerdel D, Dolnik A, Beuter F, Blatte TJ, Schmidt SA, et al. Genotyping of circulating tumor DNA in cholangiocarcinoma reveals diagnostic and prognostic information. Sci Rep. 2019;9(1):13261.
8. Carotenuto M, Sacco A, Forgione L, Normanno N. Genomic alterations in cholangiocarcinoma: clinical significance and relevance to therapy. Explor Target Antitumor Ther. 2022;3(2):200-23.
9. Tian W, Hu W, Shi X, Liu P, Ma X, Zhao W, et al. Comprehensive genomic profile of cholangiocarcinomas in China. Oncol Lett. 2020;19(4):3101-10.
10. Zheng B, Han J, Shen S, Jiang Z, Peng R, Cai J, et al. Genomic feature and potential therapeutic target for cholangiocarcinoma. Research Square; 2023.
11. Sara EY, Ramja S, Daniela S. Genomic alterations in intrahepatic cholangiocarcinoma. Hepatoma Research. 2023;9:34.
12. Sanjana NE, Shalem O, Zhang F. Improved vectors and genome-wide libraries for CRISPR screening. Nat Methods. 2014;11(8):783-4.
