# Supplementary material for: The heterogeneity of signaling pathways and drug responses in intrahepatic cholangiocarcinoma with distinct genetic mutations
Source: Cell Death Dis. 2024 Jan 11;15(1):34. doi: 10.1038/s41419-023-06406-7 (PMC10784283; doi:10.1038/s41419-023-06406-7)

Full and uncropped western blot for Figure 4

Panel A

Fgfr2---

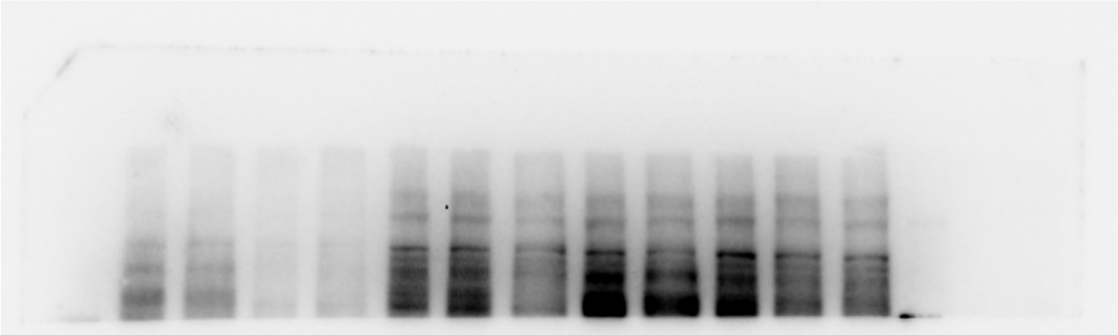

p-Fgfr---

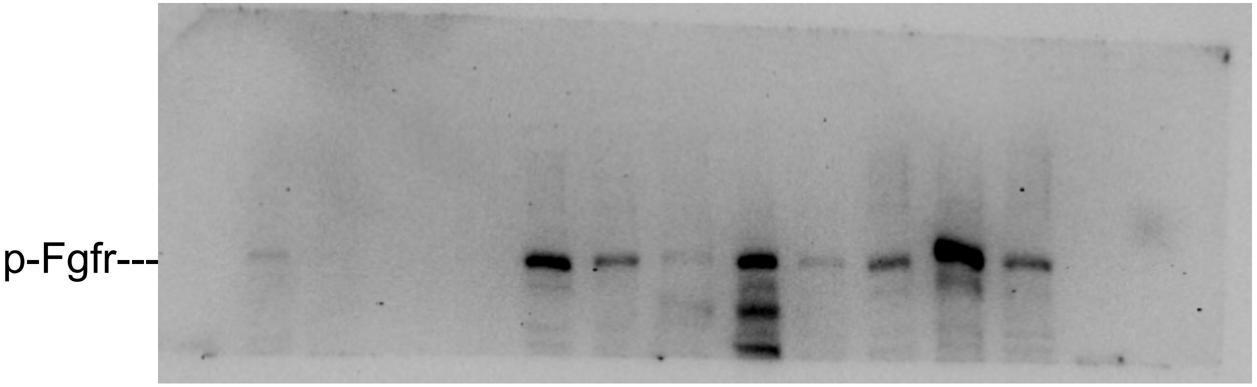

Full and uncropped western blot for Figure 4

Panel A

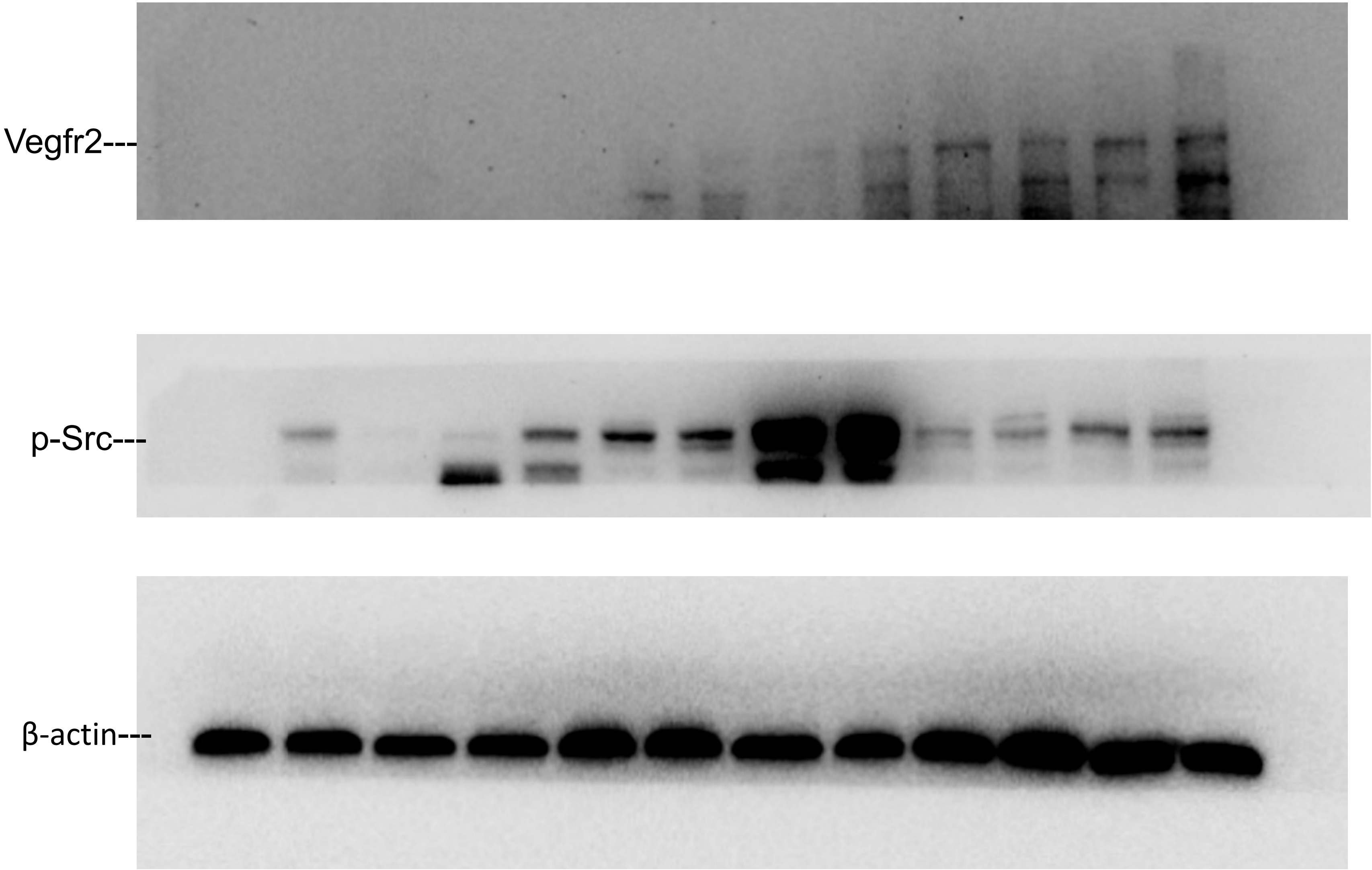

Full and uncropped western blot for Figure 4

Panel G left

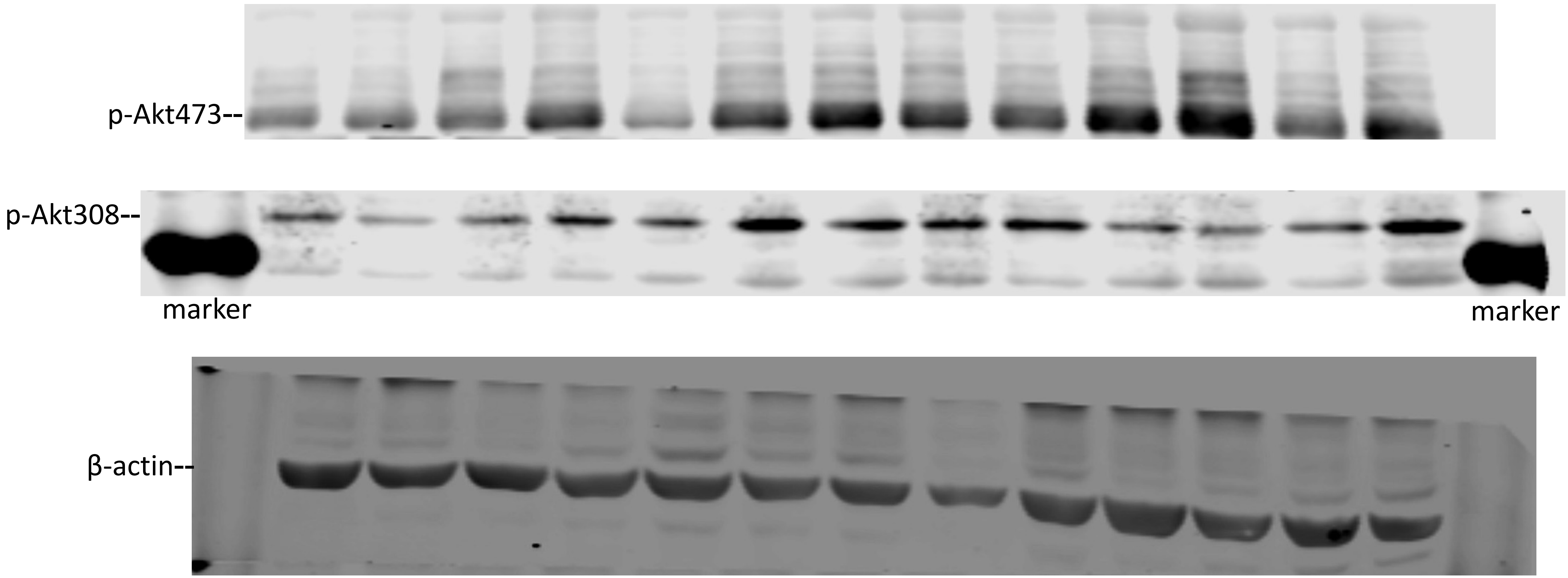

Full and uncropped western blot for Figure 4

Panel G right
Lane2 1,2,3,4,5,6,7,8 are on the figure

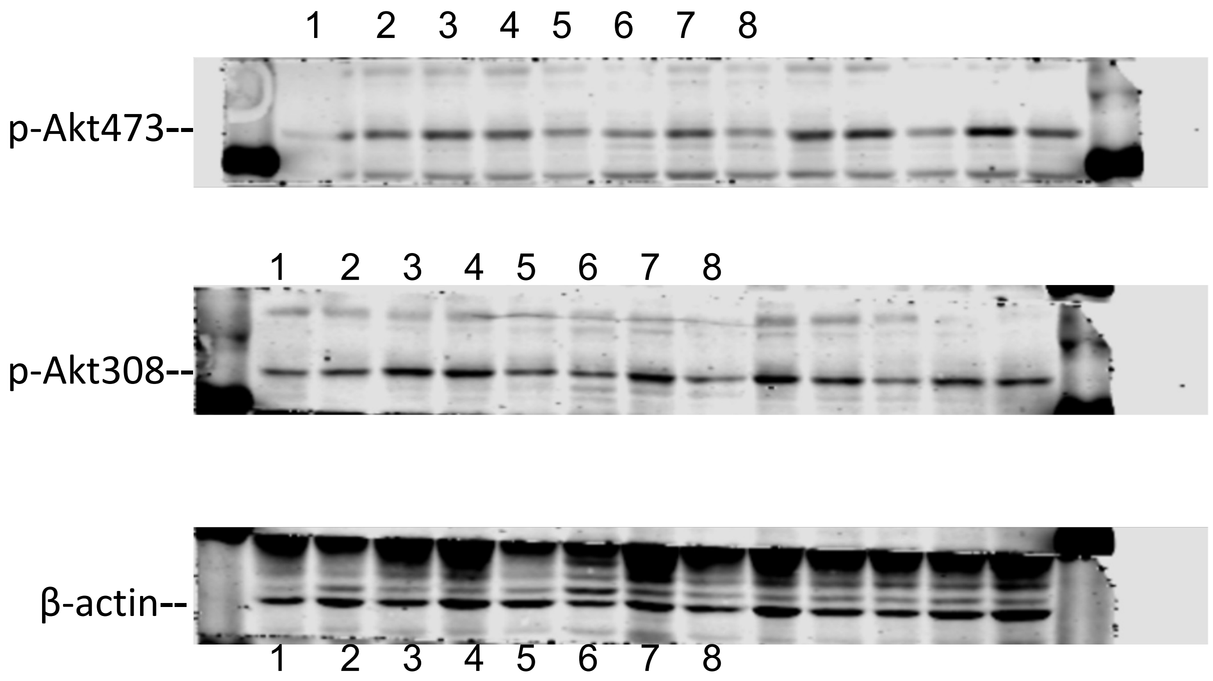

Full and uncropped western blot for Figure 7

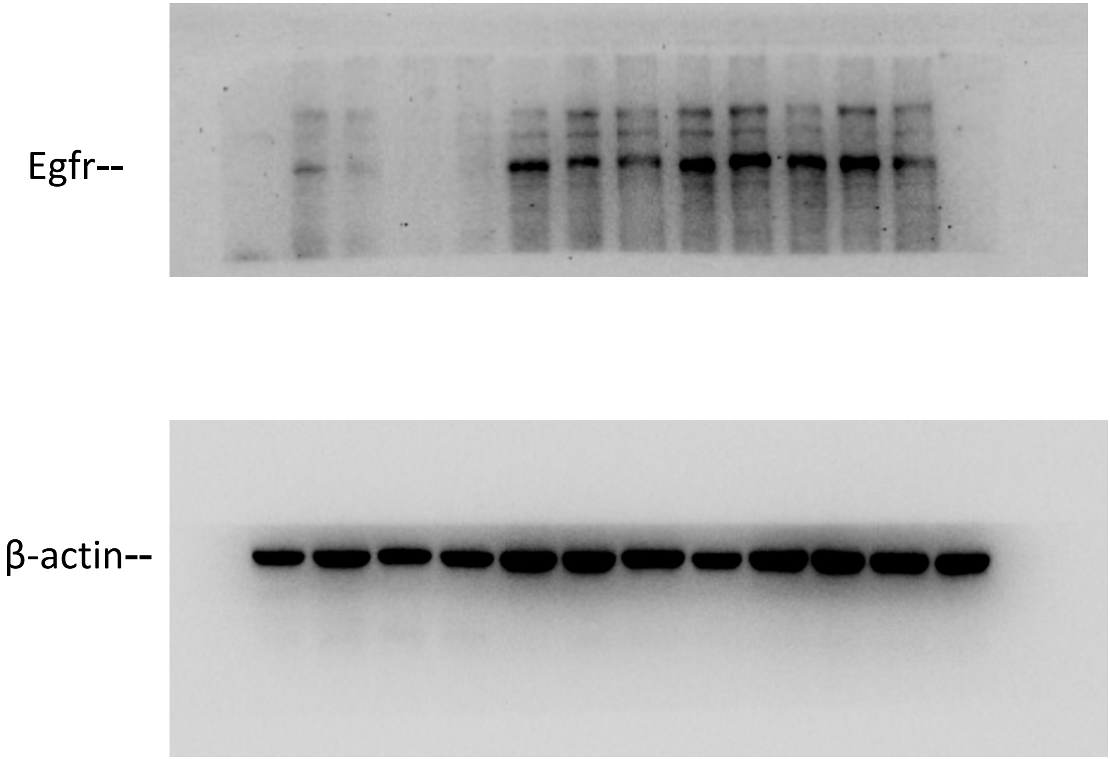

Full and uncropped western blot for Supplementary Figure 4C

QBC939 cells

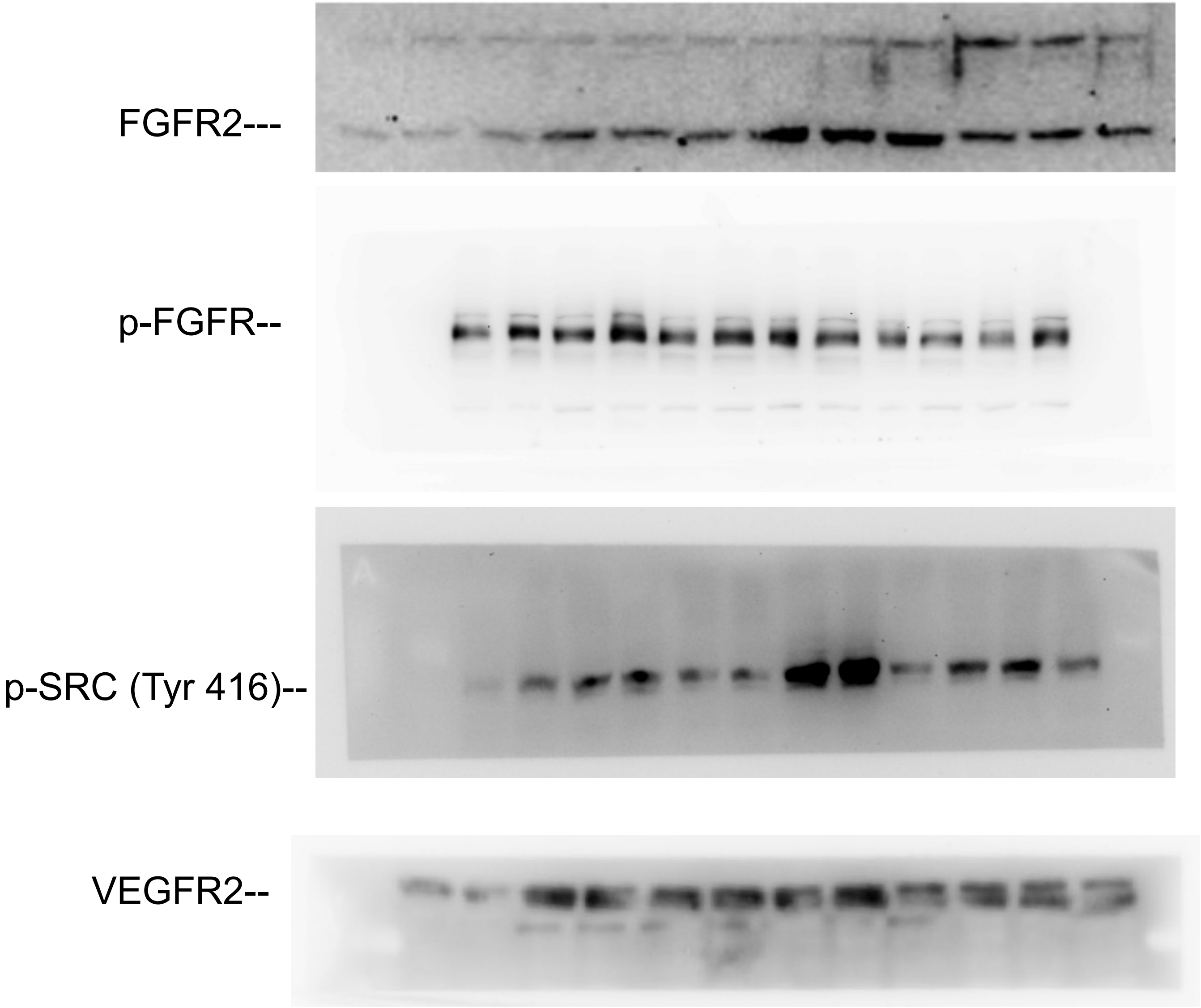

Full and uncropped western blot for Supplementary Figure 4C

QBC939 cells

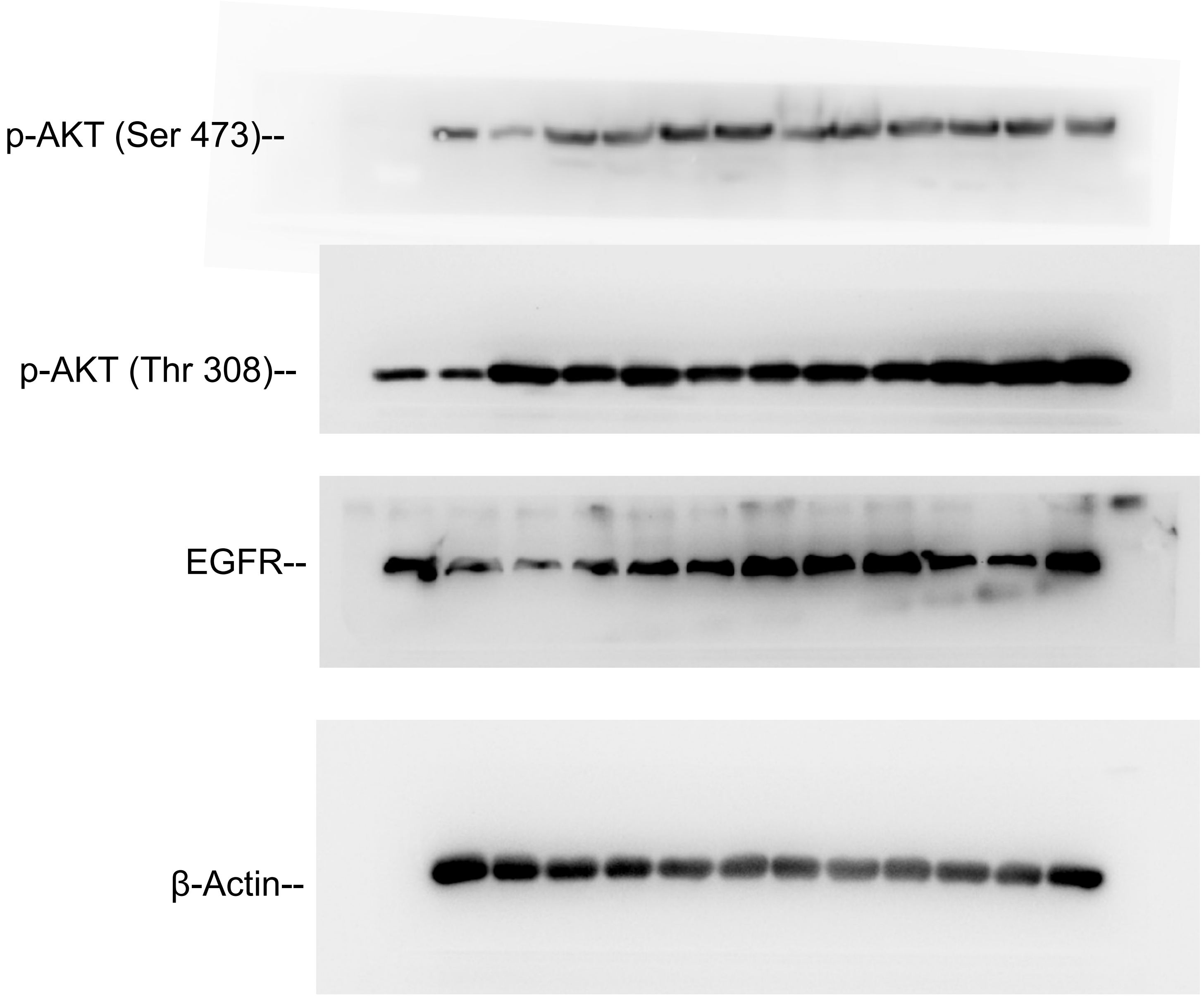

Full and uncropped western blot for Supplementary Figure 4C

HCCC9810 cells

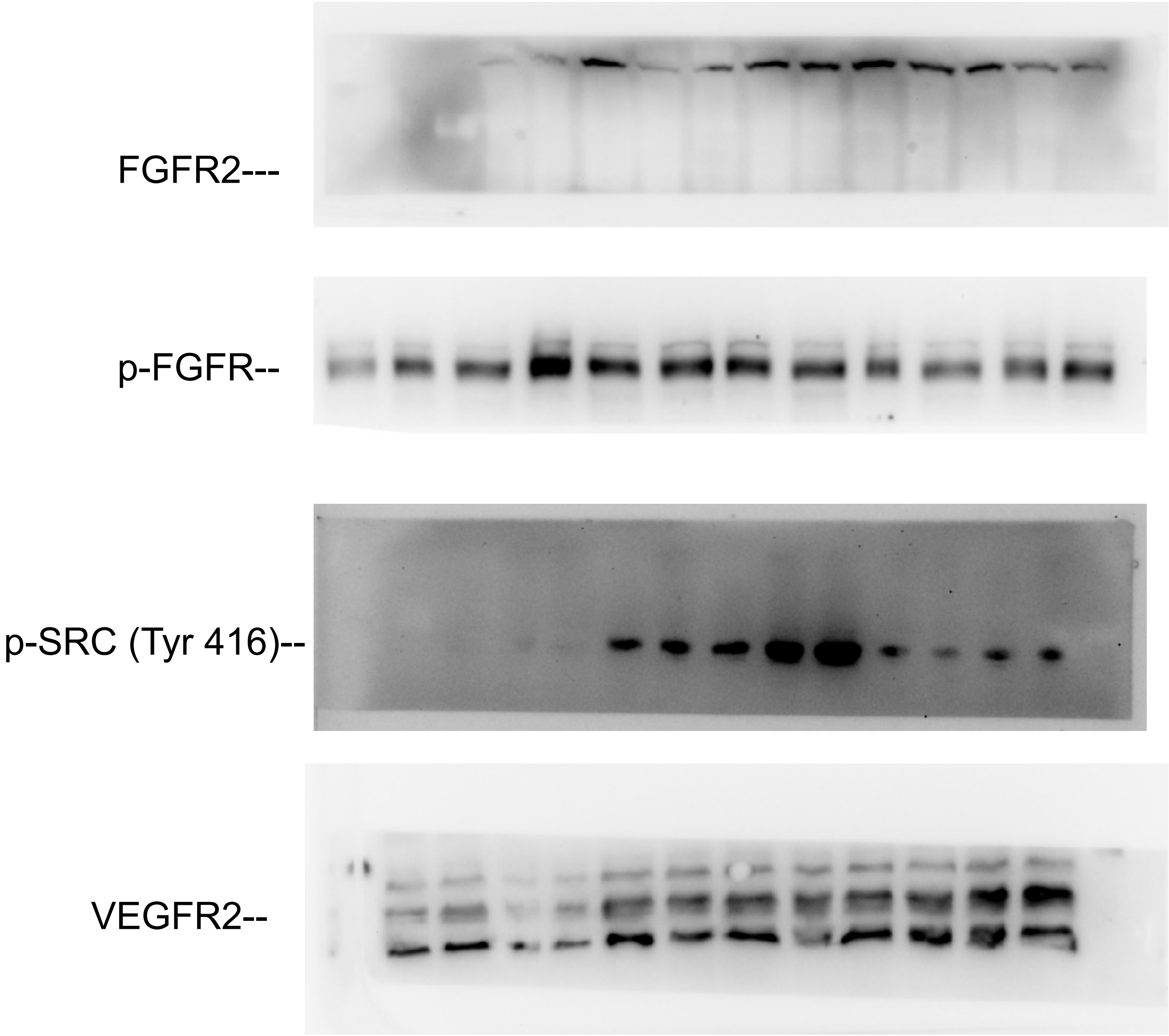

Full and uncropped western blot for Supplementary Figure 4C

HCCC9810 cells

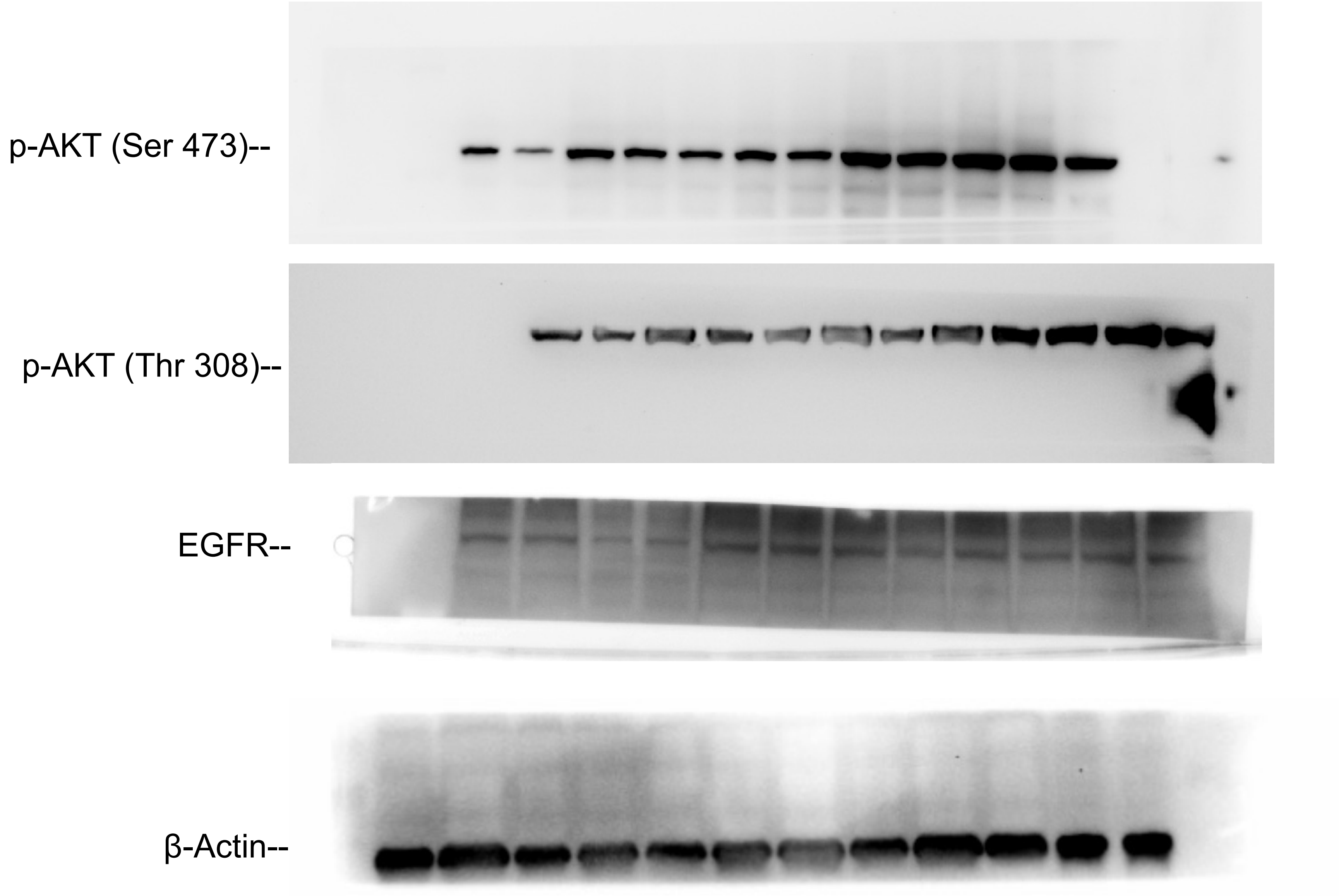

Supplement: Supplementary file 2 — Supplemental file_Uncropped WB [file 41419_2023_6406_MOESM2_ESM.pdf]
